# ﻿Further notes on the taxonomy of the land snail family Clausiliidae Gray, 1855 (Stylommatophora, Helicina) from Myanmar with description of two new species

**DOI:** 10.3897/zookeys.1160.98022

**Published:** 2023-05-03

**Authors:** Nem Sian Man, Ngwe Lwin, Chirasak Sutcharit, Somsak Panha

**Affiliations:** 1 Animal Systematics Research Unit, Department of Biology, Faculty of Science, Chulalongkorn University, Bangkok, 10330, Thailand Chulalongkorn University Bangkok Thailand; 2 Fauna and Flora International, No. 35, 3rd Floor, Shan Gone Condo, Myay Ni Gone Market Street, Sanchaung Township, Yangon, Myanmar Fauna and Flora International Yangon Myanmar

**Keywords:** Biodiversity, door snail, endemic, gastropod, limestone, systematics

## Abstract

This study presents a complete species list of the door snails inhabiting Myanmar, updated to now include 33 taxa, and provides taxonomic notes together with a re-description of the shell, radula, and genitalia for 13 species and subspecies, including *Oospiraphilippiana*, the type species of the genus *Oospira*. The snails previously treated as subspecies or synonyms of *Oospiragracilior* and *Oospiramagna* are reclassified and recognized as distinct species. The lectotype of *Oospirainsignis* has been clarified and an illustration of the original type specimen provided. A long-overlooked species, *Oospiraandersoniana*, has been collected and redescribed herein. Two new species from the limestone karsts in the Salween River Basin are introduced: *Oospiraluneainopsis* Man & Panha, **sp. nov.** and *Oospirazediopsis* Man & Panha, **sp. nov.** A synoptic view of all clausiliid taxa known from Myanmar is presented along with taxonomic information and distributional records. Photographs of the type materials for all taxa are provided for further comparison or, if unavailable, photographs of the examined specimens or the original figure from the literature.

## ﻿Introduction

Door snails are well-known land snails belonging to the family Clausiliidae Gray, 1855, and are easily recognized by their usually sinistral fusiform shell and their unique clausilial apparatus: clausilium, plicae and lamellae (Nordsieck 2007; Uit de Weerd and Gittenberger 2013). They are considered to have originated in western Eurasia during the Late Cretaceous before being dispersed worldwide, and now occur in the Ethiopian, Palearctic and Neotropical regions (Nordsieck 2007; Uit de Weerd and Gittenberger 2013). These snails have long fascinated researchers who studied their systematic and evolutionary history, particularly for European taxa (e.g., Giokas et al. 2005; Gittenberger et al. 2012; Koch et al. 2017; Fehér et al. 2018; Hausdorf 2022; Hausdorf and Neiber 2022; Sulikowska-Drozd et al. 2022).

Only two of the seven subfamilies of Clausiliidae have diversified in Southeast Asia: Phaedusinae Wagner, 1922 and Garnieriinae Boettger, 1926 (Uit de Weerd and Gittenberger 2013). Their ancestors are believed to have colonized and diversified in SE Asia independently over nearly the same period (Nordsieck 2007; Uit de Weerd and Gittenberger 2013). These door snails are ground to arboreal dwellers (tree trunks) that tend to prefer karstic-associated habitats (Stoliczka 1871; Loosjes 1953). Within Indochina, Vietnam has the highest number of reported clausiliid taxa, with ca. 90 nominal species and subspecies in 33 genera and subgenera (Nordsieck 2011; Schileyko 2011; Páll-Gergely and Szekeres 2017; Bui and Szekeres 2019); in contrast, the clausiliids of other countries in Indochina are far less well documented. This is surprising especially in the case of Myanmar, which hosts 14 terrestrial ecoregions (Olson et al. 2001) and has twice the land area of Vietnam, but which has only 31 nominal species and subspecies from four genera (Grego et al. 2021; Szekeres et al. 2021a, b).

Knowledge of Myanmarese door snails was primarily reported during the colonial period in the 19^th^ century by the pioneering western naturalists (see Pholyotha et al. 2020; Man et al. 2022 for further history review). The ‘Fauna of British India including Ceylon and Burma’ by Gude (1914) was the first, and is still the most important, publication on Myanmarese clausiliids. This early-20^th^ century reference includes 19 nominal species and subspecies belonging to ‘*Clausilia* Draparnaud, 1805’ with various subgenera and ‘sections’ (Gude 1914). Later, three additional species from northern and south-eastern Myanmar were introduced in the mid-20^th^ and early-21^st^ centuries (Likharev 1962; Nordsieck 1973, 2002a). In 2007, Nordsieck published an updated global list and proposed a reclassification of nearly all known clausiliid species. Then, in 2021, he further published the diagnostic characters, drawings and photographs from the type species and representative materials for Asian Phaedusinae (Nordsieck 2007, 2021). Regarding Myanmarese clausiliids, *Phaedusa* Adams & Adams, 1855 and *Oospira* Blanford, 1872 stand out as the most species-rich genera in this family. Recently, Grego et al. (2021) and Szekeres et al. (2021a, b) reported that the door snail diversity of Myanmar included 27 nominal species and subspecies of three genera; however, these species are mainly recognized on shell features only.

Traditionally, shell morphology employs apomorphic characters for species recognition, including lamellae development, and the number and position of palatal plicae (i.e., Nordsieck 2007, 2021; Uit de Weerd and Gittenberger 2013). Meanwhile, genital anatomy has been used to distinguish taxa when shell features alone are ambiguous (Pholyotha et al. 2020; Sutcharit et al. 2020a; Páll-Gergely and Szekeres 2020). Yet, the reproductive organs of clausiliids have received less attention, with only some scattered investigations in a few Indochinese species (Stoliczka 1871, 1873; Likharev 1962; Nordsieck 1973; Maassen and Gittenberger 2007; Páll-Gergely and Szekeres 2017). Nevertheless, recent studies on European taxa have shown that the genital organs and their internal sculpture, though highly variable, are helpful for more precise species identification (De Mattia et al. 2020, 2021; Páll-Gergely and Szekeres 2020). Lately, Hwang et al. (2022) also discussed some characters involving the genitalia and shell sculpture that should be considered for further taxonomic work on clausiliids at the species level based on a molecular analysis of the genus *Formosana* Boettger, 1877. Prior to our recent study, all former studies on Myanmarese phaedusinids were based on shell characters, except for *Oospiraphilippiana* (Pfeiffer, 1847), *O.decollata* (Likharev, 1962), and *O.malaisei* Nordsieck, 1973; the genital anatomy of these three species was described, but only the external features were reported. Therefore, there is a need for more in-depth studies of the reproductive organs of these snails and Southeast Asian clausiliids in general.

Myanmar is actively promoting knowledge of its malacofauna, especially the material obtained under the framework of a joint project between the Forest Department of Myanmar, the Fauna and Flora International (**FFI**), and the Animal Systematics Research Unit (**ASRU**) of Chulalongkorn University, Thailand. This effort has already provided information regarding the distribution, habitat, and essential morphological characters (e.g., genitalia and radula) of the Myanmarese malacofauna, including the description of several new ariophantids and helicarionids taxa (Pholyotha et al. 2020, 2022; Sutcharit et al. 2020a; Sutcharit and Panha 2021) and Streptaxidae (Sutcharit et al. 2020b; Man et al. 2022). In this context, the present study provides an up-to-date species list of the door snails from Myanmar, with taxonomic notes, and with a re-description of the shell, genitalia, and radula morphology of several species. In addition, two species from the limestone karsts in the Salween River Basin are described as new. Hopefully, this article will inspire young Myanmarese zoologists to take an interest in the land snails of their country.

## ﻿Materials and methods

### ﻿Sampling and morphological studies

**Figure 1.**
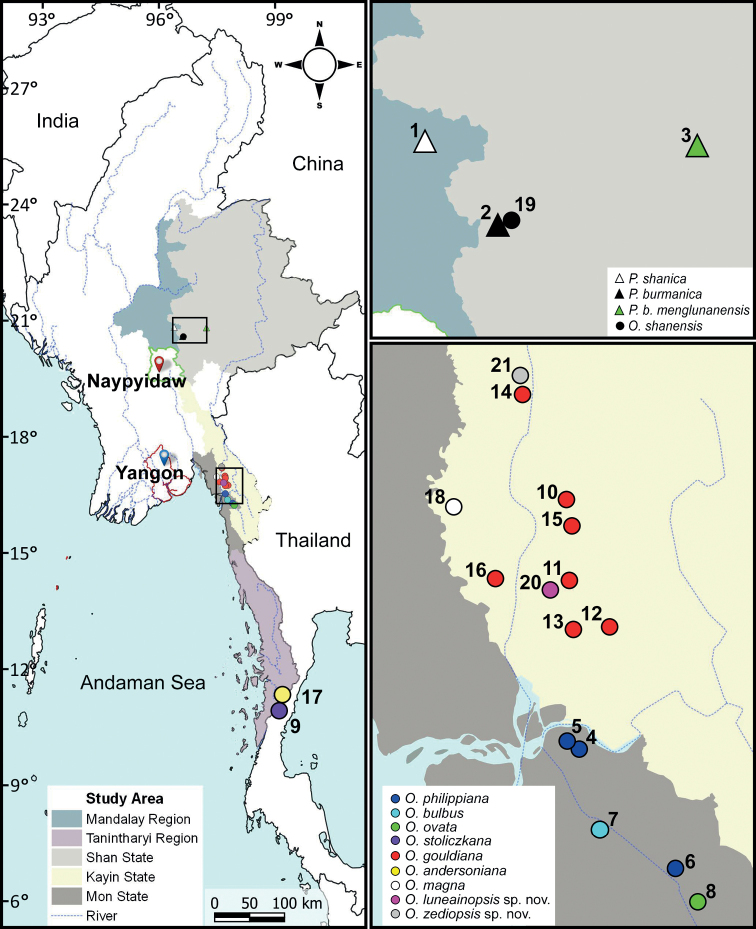
Approximate collecting localities of the *Phaedusa* species (triangles) and *Oospira* species (circles) from Myanmar examined in this study. The numbers correspond to localities listed in Table 1.

This study was conducted within the MoU (Letter No. 0092) framework between the Forest Department, Ministry of Natural Resources and Environmental Conservation and Forestry, Myanmar and FFI from 2015 to 2016. Specimens were collected throughout northern and southern Myanmar (Fig. 1; Table 1) by the ASRU members and are deposited in the Chulalongkorn University Museum of Zoology (**CUMZ**), Thailand. Living snails were photographed and euthanized following standard two-step method protocols. The snails were immersed in 5% ethanol and then preserved in 70% (v/v) ethanol for anatomical studies (American Veterinary Medical Association 2020). Species identifications were based on the original descriptions, supplemented with Gude (1914), Nordsieck (2002b, 2007), Grego et al. (2021) and Szekeres et al. (2021a, b). Specimens were also compared with available reference collections and type material.

**Table 1.** Shell measurements of clausiliid species examined in this study. The numbers listed with collection localities correspond to the map in Fig. 1. Asterisks indicate incomplete (apex broken; not decollated) shells. SH: shell height; SW: shell width.

Species no., locality no., and CUMZ no.	Number of specimens	Ranges, mean ± S.D. in mm		SH/SW ratio	Number of whorls
		Shell height	Shell width		
1. *Phaedusashanica*					
1. Pyinyaung Village, Meiktila, Mandalay (13032, 13033)	46	16.0–18.0 16.88 ± 0.87	3.0–3.4 3.25 ± 0.13	5.0–5.5 5.22 ± 0.26	11–13½
2. *Phaedusaburmanica*					
2. Myin Ma Hti, Kalaw, Shan (13034, 13035)	2	24.0–25.3 24.76 ± 0.68	5.0–5.4 5.16 ± 0.20	4.68–4.9 4.79 ± 0.10	12, 13
3. *Phaedusabockimenglunanensis*					
3. Aik Kham Cave, Taunggyi, Shan (13036)	3*	22.8, 23.9, 24.0	4.3, 4.5, 5.0	4.8–5.3 5.13±0.29	12–13
4. *Oospiraphilippiana*					
4. Saddan Cave, Mawlamyine, Mon (13037)	10	21.6–23.0 22.22 ± 0.64	7.0–8.2 7.76 ± 0.45	2.8–3.1 2.86 ± 0.12	5–6
5. Kayon Cave, Mawlamyine, Mon (13038, 13039)	21	21.0–22.6 21.96 ± 0.61	7.2–8.0 7.52 ± 0.30	2.8–3.0 2.92 ± 0.07	5–6
6. Pha Boang Cave, Mawlamyine Mon (13040¸ 13041)	24	25.5–26.6 25.95 ± 0.47	7.9–8.2 8.02 ± 0.12	3.2–3.3 3.23 ± 0.03	6–6½
5. *Oospirabulbus*					
7. Sanbel Cave, Mawlamyine, Mon (13042, 13043)	18	19.39–19.78 19.56 ± 0.17	8.00–8.55 8.30±0.19	2.28–2.42 23.57±0.05	5–5½
6. *Oospiraovata*					
8. Pathein Mountain, Mawlamyine, Mon (13044, 13045)	26	16.8–18.4 17.60±0.59	6.7–7.1 6.92 ± 0.20	2.5–2.7 2.54 ± 0.08	5–5½
7. *Oospirastoliczkana*					
9. Phra Cave, Tanintharyi (13046, 13047)	5	21.6–25 23.47 ± 1.64	6.7–7.1 6.92 ± 0.17	3.1–3.6 3.39 ± 0.22	6–6½
8. *Oospiragouldiana*					
10. Bardai Mountain, Hpa-an, Kayin (13048)	25	24.8–29.0 26.10 ± 1.69	5.7–6.2 5.90 ± 0.21	4.1–4.8 4.42 ± 0.25	10–11
11. Kaw Ka Thaung, Hpa-an, Kayin (13049)	23	25.8–27.8 26.81 ± 0.88	6–7 5.97 ± 0.32	4.3–4.9 4.50 ± 0.28	10–11
12. Lun Nga Mountain, Hpa-an, Kayin (13050)	18	24.0–27.0 26.32 ± 1.41	5.7–6.5 6.11 ± 0.34	4.0–4.9 4.31 ± 0.27	11–12
13. Sadhdan Cave, Hpa-an, Kayin (13051)	2*	25.0, 29.0	5.4, 6.2	4.63–46.7 4.65±0.34	11, 11½
14. Taung Lay Cave, Hpa-an, Kayin (13052)	30	28.1–29.2 28.50 ± 0.47	5.6–5.9 5.80 ± 0.14	4.77–5.21 4.91 ± 0.17	11½–12
15. Waiponla Mountain, Hpa-an, Kayin (13053, 13054)	4*	26.0–27.5 26.42 ±0.72	6.0–7.0 6.52±0.41	3.92–4.33 4.05±0.18	8–8½
16. Rathye Pyan Cave, Hpa-an, Kayin (13055)	1*	21.83	6.1	3.57	6
9. *Oospiraandersoniana*					
17. Phra Cave, Tanintharyi (13056, 13057)	34	18.6–22.0 19.75 ± 1.26	4.1–4.4 4.28 ± 0.13	4.3–5.0 4.60 ± 0.22	9–10
10. *Oospiramagna*					
18. Bayin Nyi Cave, Hpa-an, Kayin (13058, 13059)	4	21.8–26.3 24.68 ± 1.76	5.3–5.5 5.38 ± 0.08	4.1–4.7 4.58 ± 0.27	10–11
11. *Oospirashanensis*					
19. Nanthe Cave, Kalaw, Shan (13060, 13061)	9	24.5–26.0 25.63 ± 1.00	5.9–0.6 5.93 ± 0.60	4.0–4.6 4.34 ± 0.31	12½–13
12. *Oospiraluneainopsis* sp. nov.					
20. Zwekabin Mountain, Hpa-an, Kayin (13062, 13063)	8	18.7–21.6 20.34 ± 1.34	5.6–6.0 5.72 ± 0.16	3.3–3.9 3.50 ± 0.23	7–8
13. *Oospirazediopsis* sp. nov.					
21. Weibyan Cave, Hpa-an, Kayin (13064, 13065)	50	22.4–26.8 24.98 ± 1.43	5.7–6.8 6.25 ± 0.38	3.8–4.2 4.00 ± 0.14	10–11

**Figure 2.**
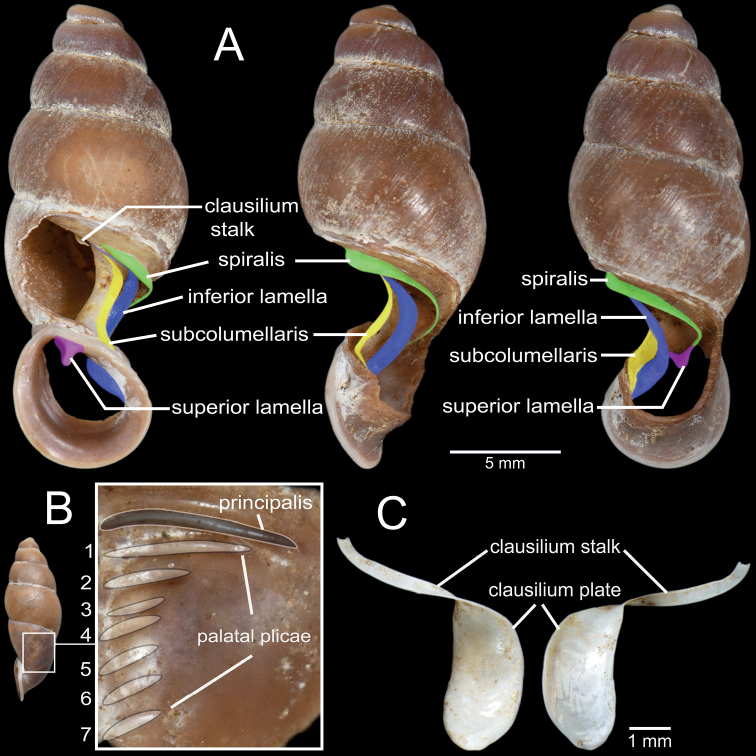
Clausilial apparatus terminology, with *Oospiraphilippiana* as a representative species **A** apertural, lateral and dorsal views from left to right, respectively, showing the lamellae **B** inside view of last whorl showing principalis and palatal plicae **C** front (left) and back (right) views of clausilium plate. Color indicates each lamella character.

Shell dimensions (height and width), whorl count, coloration and sculpture were assessed from intact adult shells. The parietal wall of the last whorl of one to three shells was removed to observe the shape, size, thickness and numbers of plicae and lamellae, and clausilium morphology. The terminology used for the clausilial apparatus and genitalia in this description is modified from Gude (1914), Loosjes (1953), and Nordsieck (2007, 2021), as shown in Fig. 2. In addition, when ethanol-preserved specimens were available, the genitalia of one to five snails of each species were dissected and observed under an Olympus SZX2-TR30 stereoscopic light microscope. The shells, clausilial apparatus and genitalia were then imaged using a digital camera (DSLR D850-Nikon) and a stereo microscope with Cell’s Imaging Software. The buccal masses were removed, and the radulae were soaked in 10% (w/v) NaOH, and then cleaned in distilled water. Radulae were photographed under scanning electron microscopy (SEM; JEOL, JSM-6610 LV).

Two nominal species names described as new to science in this work are attributed to the first and last authors (Man and Panha). Thus, a complete citation of the authorship is “Man and Panha in Man et al.”.

### ﻿Anatomical abbreviations

**at** atrium;

**bb** bursa (of bursa copulatrix);

**db** diverticulum (of bursa copulatrix);

**dpb** distal part of pedunculus (of bursa copulatrix);

**ep** epiphallus;

**fo** free oviduct;

**p** penis;

**ppb** proximal part of pedunculus (of bursa copulatrix);

**pr** penial retractor muscle;

**v** vagina;

**vd** vas deferens.

### ﻿Institutional abbreviations

**CUMZ** Chulalongkorn University Museum of Zoology, Bangkok;

**JG-C** Jozef Grego collection, Banska Bystrica, Slovakia;

**MCZ**Museum of Comparative Zoology, Harvard University, Massachusetts;

**MNHN**Muséum National ďHistoire Naturelle, Paris;

**NHMUK**The Natural History Museum, London;

**SMF**Forschungsinstitut und Naturmuseum Senckenberg, Frankfurt am Main;

**SMNH**Swedish Museum of Natural History, Stockholm;

**UF**Florida Museum of Natural History, University of Florida, Florida;

**ZISP**Zoological Institute, Russian Academy of Sciences, St. Petersburg.

## ﻿Systematics

### ﻿Family Clausiliidae Gray, 1855

**Subfamily Phaedusinae Wagner, 1922**

#### ﻿Genus Phaedusa

Taxon classificationAnimaliaStylommatophoraClausiliidae

Adams & Adams, 1855

AF320C27-ACCD-5E5B-9673-3F84F36D2C64

Clausilia (Phaedusa) Adams & Adams, 1855: 184. Boettger 1877: 54. Pfeiffer and Clessin 1881: 390. Gude 1914: 305.
Phaedusa –Thiele, 1931: 530. Zilch 1959: 389. Schileyko 2000: 599. Nordsieck 2002b: 88. Nordsieck 2007: 30.

##### Type species.

*Clausiliacorticina* Pfeiffer, 1842, by subsequent designation of Martens in Albers (1860: 275).

##### Diagnosis.

Shell fusiform, more or less turreted, spire attenuated and shell surface with fine wavy to oblique striations. Palatal plicae short mostly, middle plicae weak to developed and 3 to 7 in number. Superior lamella low, connected to or separated from spiralis, and distinct at transition to spiralis. Inferior lamella spirally ascending and ending close to superior lamella from aperture view. Subcolumellaris visible or invisible in frontal view. Clausilium plate lateral side, rounded and broad.

##### External features.

Animal covered with reticulated, dark gray skin; tentacles short; body and tail with paler color on lateral margin of foot; foot moderately elongated, posteriorly obtusely pointed.

##### Remarks.

Nordsieck (2002b) compiled all the Southeast Asian Phaedusinae and summarized their diagnostic characteristics. Although *Phaedusa* and *Loosjesia* Nordsieck, 2002 are recognized as having spirally ascending inferior lamella, *Phaedusa* possesses shorter or weaker palatal plicae with the inferior lamella more narrowly spiral; in contrast, *Loosjesia* possesses longer and more developed palatal plicae, and much more spiral inferior lamella. *Phaedusa* closely resembles the Indian *Cylindrophaedusa* Boettger, 1877 (Gittenberger et al. 2019).

In the most recent generic classifications and species accounts by Grego et al. (2021) and Szekeres et al. (2021a, b), the genus *Phaedusa* from Myanmar is comprised of eight species, of which only three species were collected and re-described herein.

#### ﻿1 Phaedusa shanica

Taxon classificationAnimaliaStylommatophoraClausiliidae

(Boettger & Ponsonby, 1888)

95D03A7A-B61A-57FD-8D1F-F870F1C24383

**Figure 3.**
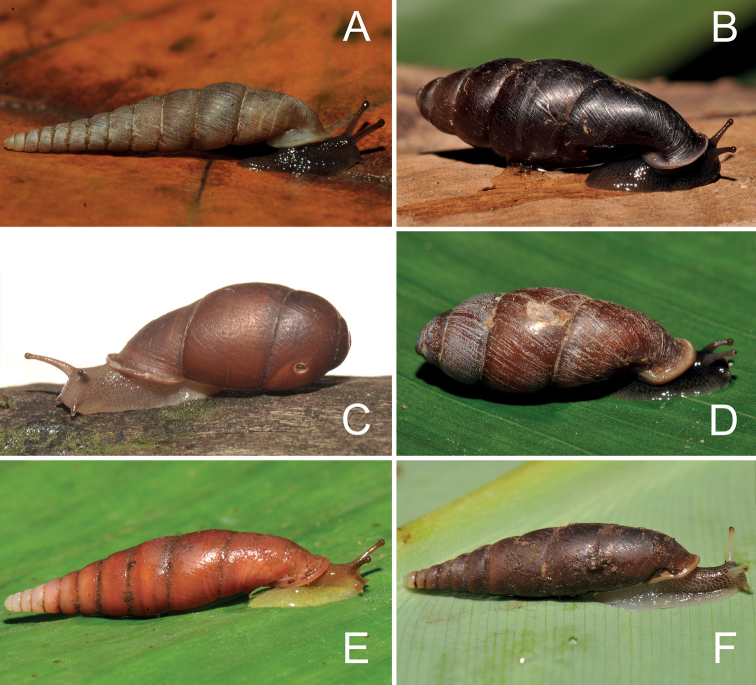
Living snails **A***Phaedusashanica* from Pyinyaung Village, Mandalay Region (SH ~ 14 mm) **B***Oospiraphilippiana* from Kayon Cave, Mon State (SH ~ 22 mm) **C***Oospirabulbus* from Sanbel Cave, Mon State (SH ~ 19 mm) **D***Oospiraovata* from Pathein Mountain, Mon State (SH ~ 16 mm) **E***Oospiragouldiana* from Kaw Ka Thuang Cave, Kayin State (SH ~ 26 mm) **F***Oospirashanensis* from Nanthe Cave, Shan State (SH ~ 25 mm).

**Figure 4.**
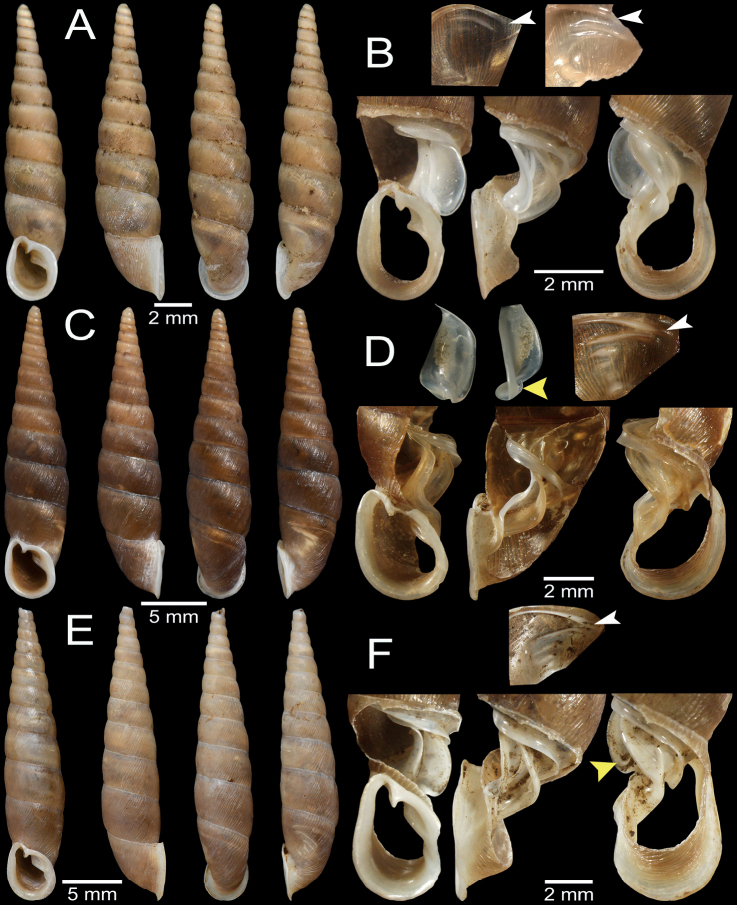
Shell and clausilial apparatus of **A, B***Phaedusashanica*, specimen CUMZ 13032 from Pyinyaung Village, Mandalay Region **A** shell **B** clausilial apparatus **C, D***Phaedusaburmanica*, specimen CUMZ 13034 from Myin Ma Hti Cave, Shan State **C** shell **D** clausilial apparatus and **E, F***Phaedusabockimenglunanensis*, specimen CUMZ 13036 from Aik Kham Cave, Shan State **E** shell **F** clausilial apparatus. White arrows indicate principalis and yellow arrows indicate clausilium hook.

**Figure 5.**
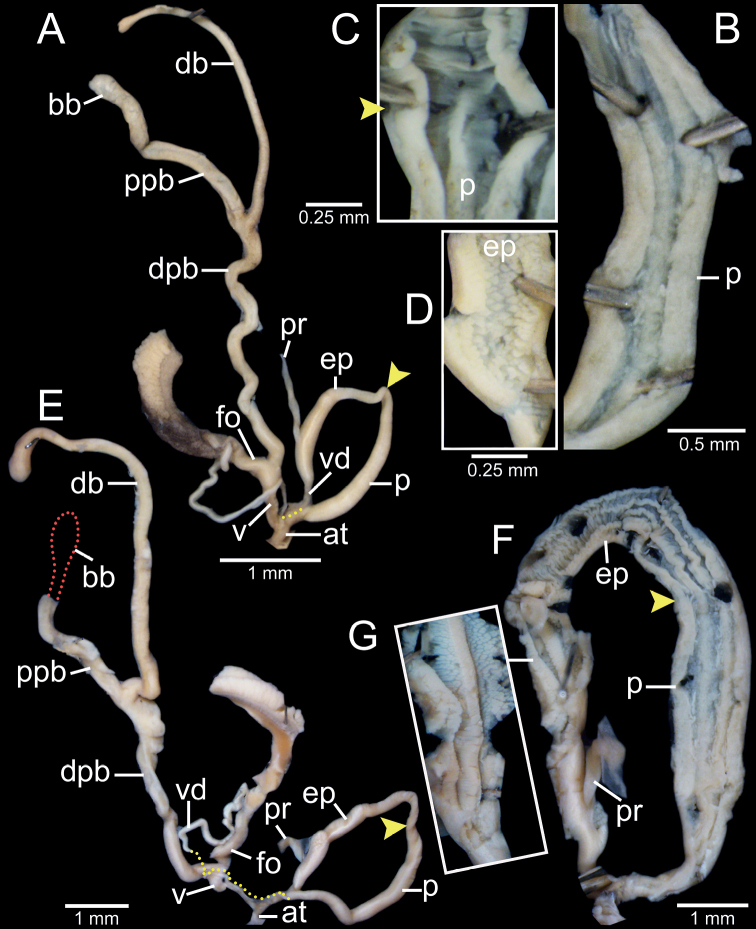
Genital anatomy of **A–D***Phaedusashanica*, specimen CUMZ 13033 **A** whole reproductive system **B** internal sculpture of penis **C** transition from penis to epiphallus **D** internal sculpture of epiphallus and **E–G***Phaedusaburmanica*, specimen CUMZ 13035 **E** whole reproductive system **F** overview internal sculpture of penis and epiphallus **G** internal sculpture of proximal epiphallus. Yellow arrows indicate approximate transitional position from penis to epiphallus.

**Figure 6.**
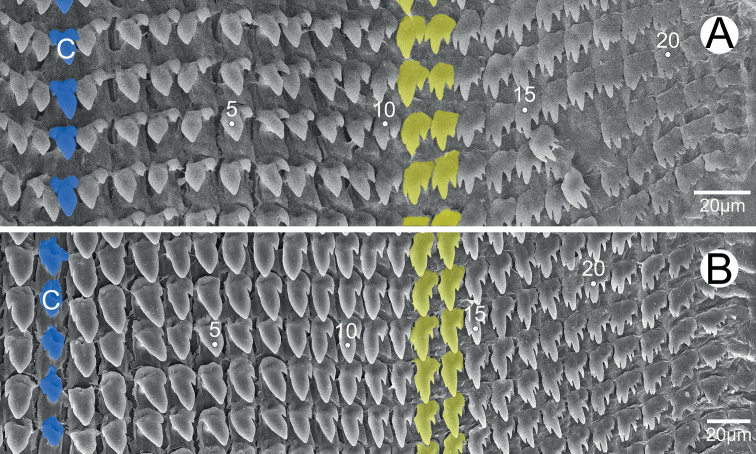
Radula morphology **A***Phaedusashanica*, specimen CUMZ 13033 and **B***Phaedusaburmanica*, specimen CUMZ 13035. Blue color indicates the central teeth row; yellow color indicates the transition from lateral to marginal teeth. ‘C’ indicates central tooth.

Figs 3A , 4A, B , 5A–D , 6A , 19K ; Tables 2 , 3

Clausilia (Pseudonenia) shanica Boettger & Ponsonby in Godwin-Austen 1888: 244. Type locality: “Shan Hills, near Pingoung, Burma, at a height of about 2500 feet” [Pinlaung Township, Shan State, Myanmar].
Clausilia [Phaedusa (Pseudonenia)] shanica–Gude 1914: 326, figs 111, 112.
Phaedusa (Phaedusa) shanica –Nordsieck 2002b: 88. Nordsieck 2007: 32.
Phaedusa shanica –Zilch 1954: 34, pl. 3, fig. 46. Nordsieck 1974: 46, fig. 6, pl. 2, fig. 6. Grego et al. 2021: 27. Szekeres et al. 2021a: 183–185, fig. 11a, b.

##### Material examined.

Limestone hills (Apache Cement Factory), Pyinyaung Village, Meiktila District, Mandalay Region, Myanmar (20°49'39.1"N, 96°23'35.1"E): CUMZ 13032 (6 shells Fig. 4A, B), CUMZ 13033 (40 specimens in ethanol), JG-C2881 (2 shells).

##### Description.

Shell fusiform, turreted, translucent, and pale yellowish brown; spire acute. Shell surface with thin and dense oblique striations; suture distinct. Whorls 11–13½, little convex, regularly growing and attenuated to apex, last whorl somewhat compressed. Aperture obliquely rounded to pear-shaped, basis rounded and narrowing towards parietal sinus; peristome slightly protruded, thickened, and expanded. Superior lamella low, continuous with spiralis, and very low at transition to spiralis to almost separated. Inferior lamella spirally ascending, close to superior lamella and ending at peristome. Subcolumellaris emerged and only visible in oblique view. Principalis running along lateral-dorsal side and anterior end visible through oblique apertural view. Palatal plicae lateral, three: first plica strong and longest; second plica very weak and close to first plica; third plica oblique from subcolumellaris and sometimes almost connected to second plica. Palatal plicae not visible without cracking shell. Clausilium plate lateral side, broad, rounded and slightly thickened at tip.

**Table 2.** Comparison of shell characters of all species from Myanmar examined in this study.

Species	Inferior lamella	Transition from superior to spiralis lamella	Subcolumellaris in oblique view	Palatal plicae number/ positions	Clausilium position / shape	Spire
*P.shanica*	spirally ascending	connect to almost separate	visible	3/ 2^nd^ weakest and lateral	lateral/ broad and round	regularly attenuated
*P.burmanica*	spirally ascending	connect or separate	visible	5/ 3^rd^ and 4^th^ weakest and lateral	lateral/ broad, round and with hook	regularly attenuated
*P.bockimenglunanensis*	spirally ascending	connect / low at transition to spiralis	visible	4–5/ reduced towards anterior and lateral	lateral/ broad, round and with hook	regularly attenuated
*O.philippiana*	steeply ascending	connected / distinctly low at transition to spiralis	visible	7–9/ reduced towards anterior and lateral	lateral/ slightly broad	blunt and regularly attenuated
*O.bulbus*	steeply ascending	connected / distinctly low at transition to spiralis	invisible	5/ nearly equal, reduced towards anterior and lateral	lateral/ narrow	round and rapidly grow
*O.ovata*	steeply ascending	connected / distinctly low at transition to spiralis	visible	6–7/ gradually reduced towards anterior and lateral	lateral/ narrow	blunt and rapidly grow
*O.stoliczkana*	steeply ascending	connected / distinctly low at transition to spiralis	visible	8–10/ gradually reduced towards anterior and lateral	lateral/ slightly broad	blunt and regularly attenuated
*O.gouldiana*	steeply ascending	connected / slightly low at transition to spiralis	invisible	5/ 1^st^ and 4^th^ or 5^th^ longest, parallelly, or irregularly oblique, and lateral	lateral/ narrow	regularly attenuated, 3 to 4 apical whorls white
*O.andersoniana*	steeply ascending	connected / indistinct at transition to spiralis	invisible	3–5 / 3^rd^ longest, parallelly oblique, and lateral	lateral/ narrow	regularly attenuated
*O.magna*	steeply ascending	connected / indistinct at transition to spiralis	invisible	4–6 / 4^th^ longest, parallelly oblique, and ventral-lateral (almost horizontal)	ventral/ narrow	regularly attenuated, 3 to 4 apical whorls white
*O.shanensis*	steeply ascending	connected / indistinct at transition to spiralis	visible	5 / 4^th^ longest and curved, parallel, and ventral-lateral (almost horizontal)	lateral/ broad, round and with hook	regularly attenuated
*O.luneainopsis* sp. nov.	steeply ascending	connected / slightly low at transition to spiralis	invisible	4 / 4^th^ longest, parallelly oblique, and lateral	lateral/ narrow, slightly pointed at tip	blunt and regularly attenuated
*O.zediopsis* sp. nov.	steeply ascending	connected / slightly low at transition to spiralis	invisible	4–5 / 4^th^ longest, parallelly oblique, and lateral	lateral/ narrow	regularly attenuated, 4 to 5 apical whorls white

**Table 3.** Comparison of genital characters of all species from Myanmar examined in this study.

Species	Penis / epiphallus	Internal sculpture of penis	Internal sculpture of epiphallus	Distal and proximal lengths of pedunculus
*P.shanica*	muscular, large / ca. same diameter with penis	smooth surface of longitudinal folds	papillae arranged in oblique rows	distal longer than proximal
*P.burmanica*	muscular, large / ca. same diameter with penis	wavy surface of longitudinal folds	wavy longitudinal folds and papillae arranged in oblique rows	distal longer than proximal
*O.philippiana*	muscular, large / narrower than penis	chevron-shaped and smooth surface of longitudinal folds	elevated papillae arranged in oblique rows	distal shorter than proximal
*O.bulbus*	muscular, large / narrower than penis	chevron-shaped and little wavy surface of longitudinal folds	elevated papillae arranged in oblique rows	distal ca. same as proximal
*O.ovata*	muscular, large / narrower than penis	V-shaped rows and smooth surface of longitudinal folds	rounded papillae arranged in oblique rows	distal longer than proximal
*O.stoliczkana*	muscular, large / narrower than penis	nearly V-shaped and oblique smooth surface of longitudinal folds	elevated papillae arranged in nearly oblique rows	distal longer than proximal
*O.gouldiana*	moderately muscular, slender / nearly same or narrower diameter than penis	smooth surface of longitudinal folds	irregular corrugated folds and papillae arranged in oblique rows	distal ca. same as proximal
*O.andersoniana*	moderately muscular, slender / nearly same or narrower diameter than penis	smooth surface of longitudinal folds	scattered papillae arranged obliquely to irregularly spaced rows	distal longer than proximal
*O.magna*	moderately muscular, slender / nearly equal diameter with penis	smooth surface of longitudinal folds	papillae arranged in oblique rows	distal shorter than proximal
*O.shanensis*	muscular, large / ca. same diameter with penis	corrugated surface of longitudinal folds	reticulated pattern	distal longer than proximal

***Genitalia*** (*n* = 5). Atrium (at) short; penis (p) muscular, cylindrical, and gradually narrower towards epiphallus. Epiphallus (ep) muscular, cylindrical, ca. same as penis length and size, and gradually widening to proximal end. Penial retractor muscle (pr) attached proximally to epiphallus. Vas deferens (vd) slender, and shortly bounded at atrium and penis junction (Fig. 5A). Internal sculpture of penis smooth-surfaced and with 3 to 5 longitudinal folds (Fig. 5B); at transition from penis to epiphallus with irregularly corrugated and wrinkled folds that randomly split and merge (Fig. 5C). Internal sculpture of epiphallus with large papillae arranged in oblique rows (Fig. 5D).

Vagina (v) slender at junction of atrium, then bulging at connection of free oviduct (fo) and distal part of pedunculus (dpb), and almost as long as free oviduct. Distal part of pedunculus large, long, and basally stouter; diverticulum (db) slender, nearly equal to dpb length; proximal part of pedunculus (ppb) large, long, measuring ~ 3/4 of diverticulum length, and bursa copulatrix (bb) ovoid (Fig. 5A).

***Radula*.** Each row contains ~ 47 teeth with half-row formula: central–(lateral)–marginal teeth (1–(11–12)–23). Central tooth symmetrically tricuspid; mesocone large with pointed tip; ectocones triangular and located near the base. Lateral teeth asymmetrically bicuspid with small and pointed ectocone located at base; endocone large with pointed tip. Marginal teeth with asymmetrical tricuspids starting at approximately tooth number 11 or 12; mesocone large with dull tip; endocone and ectocone with small and pointed tips. Marginal teeth gradually reduced in size and becoming multicuspid marginally (Fig. 6A).

##### Distribution.

*Phaedusashanica* is collected from probably nearby the type locality and likely restricted in Myanmar. It appears to be quite abundant in the limestone hills based on the high numbers collected in our samplings.

##### Remarks.

*Phaedusashanica* is very similar to *P.theobaldi* (Blanford, 1872) in having two or three palatal plicae which tend to connect at the second and third plicae. *Phaedusatheobaldi* (Fig. 19M) can be distinguished by its less turreted shell, coarser striations, darker horny color, superior lamella and spiralis separated, and not emerged subcolumellaris. These two species are difficult to distinguish at first sight; additional materials from the type locality and the genitalia of *P.theobaldi*, are required to elucidate the relationship between these two species.

Our specimens slightly differ from the type specimen in having three separated palatal plicae, or sometimes second and third plicae well developed and connected (Fig. 4B). According to Nordsieck’s (1974: fig. 6) drawing, the lectotype SMF 62260 has only two almost connected first and second palatal plicae, while the probable paralectotype as stated in Gude (1914: fig. 112) has palatal plicae pattern similar to the specimens examined herein. Therefore, we assume that the distinct or indistinct second plica might be attributed to morphological variation. Shell variations among the examined materials for *P.shanica* were observed, including slender to regular fusiform shell, oblique to subquadrate aperture, and the third plica may be well developed and reach to the second plica (Fig. 4B).

In addition, *Phaedusalypra* (Mabille, 1887) from Shan State also resembles this species in shell form; however, *P.lypra* has a blunter spire, less turreted shell, and wider aperture sinus (Szekeres et al. 2021a: fig. 10f). *Phaedusaburmanica* (Gude, 1914) and *P.bockimenglunanensis* (Luo, Cheng & Zhang, 1998) from Shan State differ from *P.shanica* in having a larger shell, five distinct palatal plicae that can be seen from outside of shell, wavier and coarser shell surface, and clausilium with a hook (Fig. 4). Regarding the genitalia, *P.shanica* possesses more simple and smooth longitudinal folds and reticulated papillae in the epiphallus, while *P.burmanica* has strongly wavy, irregularly dense, and crowded longitudinal folds along the epiphallus with a distinct fold near the vas deferens (Fig. 5).

#### ﻿2 Phaedusa burmanica

Taxon classificationAnimaliaStylommatophoraClausiliidae

(Gude, 1914)

39C456FE-0B05-5B3F-BE22-9C90A1930B69

Figs 4C, D , 5E–G , 6B , 18F ; Tables 2 , 3

Clausilia [Phaedusa (Euphaedusa)] burmanica Gude, 1914: 311, fig. 106. Type locality: “Burma, Tonghu” [Taungoo District, Bago Region, Myanmar].
Phaedusa (Phaedusa) burmanica –Nordsieck 2002b: 88. Nordsieck 2007: 32.
Phaedusa burmanica –Nordsieck 1974: 46, fig. 5, pl. 2, figs 3, 4. Grego et al. 2021: 27. Szekeres et al. 2021a: 181, fig. 10a.

##### Material examined.

Myin Ma Hti Cave, Kalaw City, Shan State, Myanmar (20°35'26.1"N, 96°36'42.6"E): CUMZ 13034 (2 shells + 1 incomplete shell; Fig. 4C, D), CUMZ 13035 (1 specimen in ethanol).

##### Description.

Shell fusiform, turreted, translucent, and pale chestnut-brown; spire acute. Shell surface glossy with thin and dense striations; suture distinct. Whorls 12–13, little convex, regularly growing and attenuated to apex; last whorl compressed. Aperture vertically subquadrate, basis rounded and narrowing towards parietal sinus; peristome slightly protruded, thickened, and expanded. Superior lamella low, continuous with spiralis, and very low at transition to spiralis. Inferior lamella spirally ascending, close to superior lamella and ending at peristome. Subcolumellaris emerged, and visible in oblique view. Principalis running along lateral-dorsal side and anterior end visible through oblique apertural view. Palatal plicae lateral, distinctly five: first plica strong, longest, and nearly connected to principalis anteriorly; second and fifth plicae almost equal and slightly strong; third and fourth plicae weakest and almost not visible. Palatal plicae visible through translucent shell. Clausilium plate lateral side, broad, rounded and with hook.

***Genitalia*** (*n* = 1). Atrium (at) short and slender; penis (p) muscular, cylindrical, broadest at middle part and gradually narrower towards epiphallus. Epiphallus (ep) muscular, cylindrical, ca. same length and size as penis, gradually enlarging to proximal end. Penial retractor muscle (pr) attached proximally to epiphallus. Vas deferens (vd) slender, and shortly bounded at atrium and penis junction (Fig. 5E). Internal sculpture of penis smooth-surfaced and slightly wavy with ca. four longitudinal folds (Fig. 5F). Internal sculpture of epiphallus generally defined as two parts: region near penis with wavy longitudinal folds, then gradually becoming more densely crowded towards proximal epiphallus; region near vas deferens with strong folds and papillae arranged in oblique rows (Fig. 5G).

Vagina (v) slender at junction of atrium, then bulging at connection of free oviduct (fo) and distal part of pedunculus (dpb), and almost as long as free oviduct. Distal part of pedunculus large, long, basally stouter, and broadest near entrance of diverticulum; diverticulum (db) large, as long as dpb; proximal part of pedunculus (ppb) large, measuring ~ 1/2 diverticulum length, and bursa copulatrix (bb) ovoid (Fig. 5E).

***Radula*.** Each row contains ~ 51 teeth with half-row formula: central–(lateral)–marginal teeth (1–(13–14)–25). Central tooth small, symmetrically tricuspid; mesocone large with dull tip; ectocones very small. Lateral teeth asymmetrically bicuspid: endocone large and with dull tip; ectocone very small, pointed tip and located near the base. Marginal teeth tricuspid starting at approximately tooth number 13 or 14; mesocone long and dull tip; endocone very small and located near tip of mesocone; ectocone small with pointed tip. Marginal teeth gradually becoming polycuspid towards radula margin (Fig. 6B).

##### Distribution.

Apart from the type locality, this species is widely known from Bhamo, Kachin State (Gude 1914) to Shan State and the Mandalay Region in Myanmar (Grego et al. 2021).

##### Remarks.

*Phaedusabockimenglunanensis* from Shan State can be distinguished from this species by its inferior lamella less spirally ascending, palatal plicae longer, obliquely arranged and more spaced between them, shell surface finer and more curved striations, aperture vertically quadrate, and subcolumellaris distinctly visible in frontal view. *Phaedusabockithompsoni* Grego & Szekeres, 2021 also differs from this species in having a more vertical aperture, acute spire, and palatal plicae more on dorsal side (see Szekeres et al. 2021a: fig. 9f).

Variation was observed among the examined specimens in having the superior lamella and spiralis either separated or connected.

#### ﻿3 Phaedusa bocki menglunanensis

Taxon classificationAnimaliaStylommatophoraClausiliidae

(Luo, Chen & Zhang, 1998)

3C30F0D1-71AD-53F2-86DB-E8F13DA49C7F

Figs 4E, F , 19E ; Table 2

Hemiphaedusa menglunanensis Luo et al., 1998: 34, fig. 2. Type locality: Menglun, Mengla County, Xishuangbanna Prefecture, Yunnan Province, China. Chen and Zhang 1999: 162, pl. 5, fig. 1a, b. Chen et al. 2016: 111, fig. 2–52a.
Phaedusa (Phaedusa) bocki menglunanensis –Nordsieck 2007: 32.
Phaedusa bocki menglunanensis –Szekeres et al. 2021a: 180, 181, fig. 9e. Szekeres et al. 2021b: 46.

##### Material examined.

Aik Kham Cave, Taunggyi City, Shan State, Myanmar (20°49'7.0"N, 97°13'42.0"E): CUMZ 13036 (3 incomplete shells, Fig. 4E, F).

##### Description.

Shell fusiform, translucent, and pale yellowish brown; spire regularly acute. Shell surface with fine and coarse striations; suture wide and distinct. Whorls 12 and 13 nearly flattened, regularly increasing, and attenuated to apex. Aperture vertically subquadrate, basis rounded and narrowing towards parietal sinus; peristome protruded, expanded and little reflexed. Superior lamella low, continuous with spiralis, and low at transition to spiralis. Inferior lamella spirally ascending, slightly horizontal before reaching peristome and close to superior lamella. Subcolumellaris emerged, ending at peristome margin, and clearly visible in frontal view. Principalis running along lateral-dorsal side and anterior end visible through oblique apertural view. Palatal plicae lateral, four or five: first plica distinct, longest, and nearly connected to principalis anteriorly; second plica ~ 1/2 of first plica length and remaining plicae oblique, equally spaced, and shorter. Palatal plicae visible through translucent shell. Clausilium plate lateral side, broad, rounded, and with hook.

##### Distribution.

*Phaedusabockimenglunanensis* was originally described from Yunnan Province, China, which is near the borders with Vietnam and Laos. It was recently found in Shan State, along the Mekong River near the border with Laos (Szekeres et al. 2021a).

##### Remarks.

No preserved specimens were found for examination of the genitalia. This subspecies and samples of *P.bockithompsoni* collected from the same geographical range have a similar shell morphology, but *P.bockithompsoni* has invisible subcolumellaris in frontal view, blunter and turreted spire, and whorls after antepenultimate whorl rapidly attenuated (see Szekeres et al. 2021a: fig. 9f). However, the holotype of *P.bockithompsoni* from Thailand possesses a subcolumellaris that is visible in frontal view, and four to seven plicae that become shorter and somewhat diffuse towards the base (Szekeres et al. 2021b: fig. 2d). More materials are required for comparison to determine whether the differentiating characters are consistently distinct between these two subspecies.

#### ﻿Genus Oospira

Taxon classificationAnimaliaStylommatophoraClausiliidae

Blanford, 1872

12200D19-A0B0-5A53-B9A6-FFB30E63ABE8

Clausilia (Oospira) Blanford, 1872: 205. Boettger 1877: 64. Gude 1914: 332.
Phaedusa (Oospira) –Zilch 1959: 389, 390.
Oospira –Schileyko 2000: 581. Nordsieck 2002b: 86. Nordsieck 2007: 23.

##### Type species.

*Clausiliaphilippiana* Pfeiffer, 1847, by original designation.

##### Diagnosis.

Shell short to long fusiform, shell surface smooth to with striated ridges, spire blunt to attenuated, whorls few to numerous (6–13). Palatal plicae short to long, mostly developed and 3–10 in number. Superior lamella developed, usually connected to spiralis, distinct or indistinct at transition to spiralis. Inferior lamella steeply ascending and somewhat ending distant from superior lamella in aperture view. Subcolumellaris usually invisible in frontal view; clausilium plate lateral to ventral side and usually narrow.

##### External features.

Living animals possess reticulated skin; dark gray head; short tentacles; body and tail pale to dark brownish; foot moderately elongated, posterior nearly rounded to bluntly pointed.

##### Remarks.

*Oospira* is the most species-rich genus of the Phaedusinae, and more than a hundred species have been reported from a broad range in Southeast Asia (MolluscaBase 2022). Nordsieck (2002b) united two previously recognized genera, *Acrophaedusa* Boettger, 1877 and *Pseudonenia* Boettger, 1877 to form *Oospira* based on their shared steeply ascending inferior lamella, normal clausilium plate, and mostly palatal plicae form. Perhaps this grouping causes *Oospira* to become widely diverged in shell shape from ovate to fusiform, dark brown to pink, and different aperture shapes which is thought to be a homoplasy. A phylogenetic analysis of phaedusinids genera from Japanese and some southeast Asian taxa, especially from Vietnam, was not in line with traditional taxonomy probably due to parallel or convergent evolution (Motochin et al. 2017; Mamos et al. 2021). However, none of the Indochinese taxa, including *Oospira*, were explored in this phylogenetic work.

The genera *Loosjesia* from Thailand, *Messageriella* Páll-Gergely & Szekeres, 2017 and *Castanophaedusa* Páll-Gergely & Szekeres, 2017 from Vietnam, *Musaphaedusa* Nordsieck, 2018 from Laos, and *Oospira* have a similar shell morphology, especially in the form of the palatal plicae. However, *Loosjesia* can readily be differentiated by its predominantly curved basal inferior lamella with a long transverse palatal plica and clearly visible subcolumellaris (Loosjes 1953; Nordsieck 2002b). *Messageriella* is distinguished by its marginally ending inferior lamella and subcolumellaris, palatal plicae non-parallel and ventral (Páll-Gergely and Szekeres 2017); similarly, *Castanophaedusa* has a rhomboidal aperture which extends to the columellar side and sharply bent basis, marginal and strongly emerged subcolumellaris, palatal plicae ventral and parallel to the principalis (Páll-Gergely and Szekeres 2017). Meanwhile, *Musaphaedusa* has strong sutural papillae, principalis very long, palatal plicae ventral (only two) and strong rib-like striations on the shell surface (Nordsieck 2018).

Regarding the reproductive anatomy of this group, *Oospirapenangensis* (Stoliczka, 1873) and *O.philippiana* were the first species examined for their genitalia (Stoliczka 1871, 1873). At present, seven species (including *O.decollata* and *O.malaisei* from Myanmar) have been investigated (Loosjes 1953; Likharev 1962; Nordsieck 1973; Maassen and Gittenberger 2007; Páll-Gergely and Szekeres 2017).

Based on shell morphology, we have divided *Oospira* from Myanmar into two species groups comprised of those with (i) short and ovate fusiform shells and (ii) long and slender fusiform shells. The short and ovate fusiform group includes the species believed to be *Oospira* s.s. (type species *Clausiliaphilippiana* Pfeiffer, 1847), while the long and slender fusiform group assumed to be the species previously known as ‘*Pseudonenia* Boettger, 1877’ (type species *Clausiliajavana* Pfeiffer, 1841).

###### ﻿*Oospira* species group with short and ovate fusiform shell

In Myanmar, this group is comprised of five species, four of which were collected and examined, while *O.vespa* (Gould, 1856) was not.

#### ﻿4 Oospira philippiana

Taxon classificationAnimaliaStylommatophoraClausiliidae

(Pfeiffer, 1847)

BE7342AB-027F-56E9-BBE3-B1A9D35183F7

**Figure 7.**
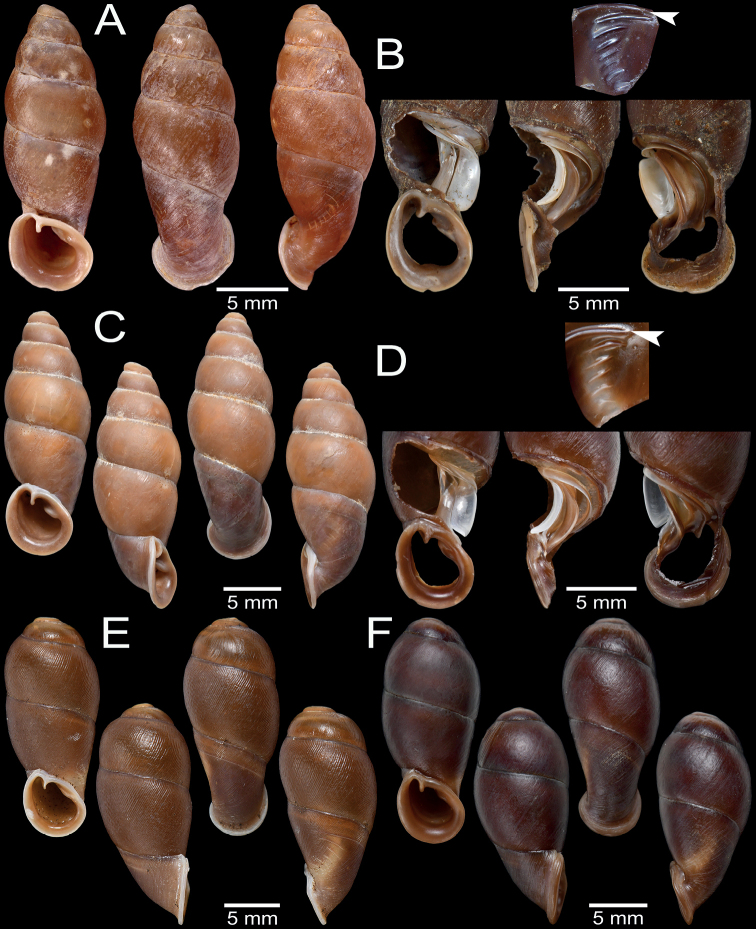
Shell and clausilial apparatus of **A–C***Oospiraphilippiana***A** possible syntype SMF 62294/1 from Burma **B** clausilial apparatus, specimen CUMZ 13040 from Pha Boang Cave, Mon State **C** shell, specimen CUMZ 13037 from Saddan Cave, Mon State and **D–F***Oospirabulbus*, specimen CUMZ 13042 from Sanbel Cave, Mon State **D** clausilial apparatus **E, F** shells. White arrow indicates principalis.

**Figure 8.**
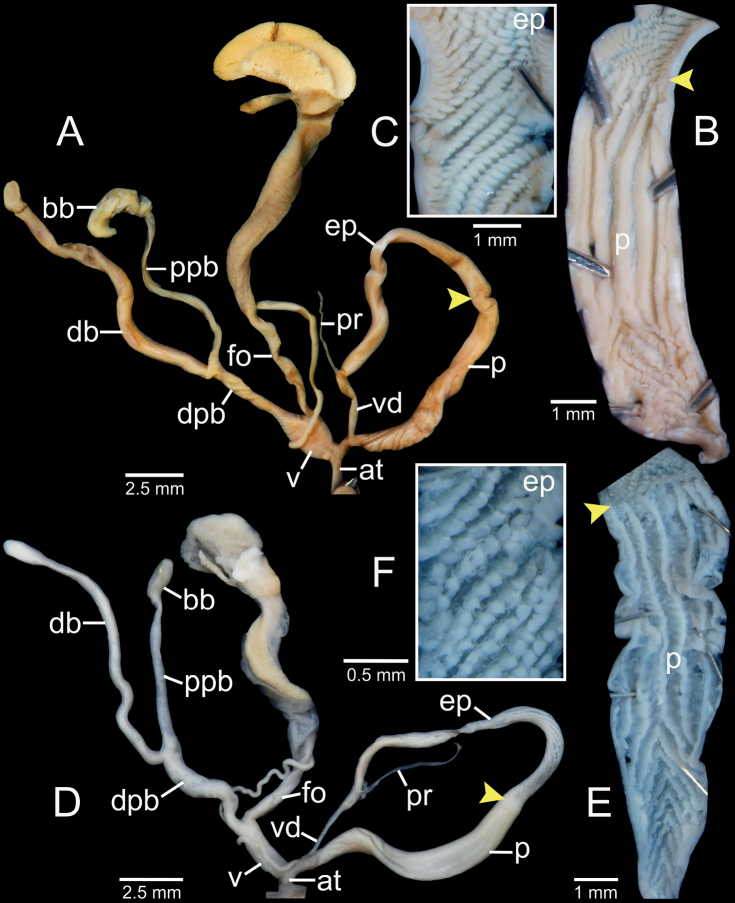
Genital anatomy of **A–C***Oospiraphilippiana*, specimen CUMZ 13039 **A** whole reproductive system **B** internal sculpture of penis **C** internal sculpture of epiphallus and **D–F***Oospirabulbus*, specimen CUMZ 13043 **D** whole reproductive system **E** internal sculpture of penis **F** internal sculpture of epiphallus. Yellow arrows indicate approximate transitional position from penis to epiphallus.

Figs 2 , 3B , 7A–C , 8A–C , 15A , 19H ; Tables 2 , 3

Clausilia philippiana Pfeiffer, 1847: 69. Type locality: “Mergui in imperio Birmanorum” [near Myeik, Tanintharyi Region, Myanmar]. Küster 1850: 100, pl. 11, figs 7–9. Pfeiffer 1868: 409. Hanley and Theobald 1874: 48, pl. 118, fig. 10. Sowerby 1875: Clausilia pl. 4, species 35.
Clausilia (Phaedusa) philippiana –Adams and Adams 1855: 184. Pfeiffer 1856: 180. Martens 1860: 275. Stoliczka 1871: 174, pl. 6, figs 7‒10.
Clausilia (Oospira) philippiana –Blanford 1872: 205, pl. 9, fig. 14. Nevill 1878: 182. Kobelt 1880: 289, pl. 86, fig. 29. Pfeiffer and Clessin 1881: 392. Tapparone-Canefri 1889: 329. Gude 1914: 333.
Clausilia [Phaedusa (Oospira)] philippiana–Boettger 1878: 56.
Oospira (Oospira) philippiana –Nordsieck 2002b: 86. Nordsieck 2007: 24.
Oospira philippiana –Nordsieck 2021: 52, pl. 1, fig. 8. Szekeres et al. 2021a: 175, fig. 8c. Szekeres et al. 2021b: 45.

##### Material examined.

Possible syntype SMF 62294/1 ex. H. Dohrn collection from Burma: [Mergui] (Fig. 7A). Saddan Cave, ~ 600 m south of Kayon Cave, Mawlamyine Township, Mawlamyine District, Mon State, Myanmar (16°31'42.8"N, 97°43'2.1"E): CUMZ 13037 (10 adults + 5 juveniles, Fig. 7C). Kayon Cave [formerly called Farm Caves] ~ 10 km from Mawlamyine Township, Mawlamyine District, Mon State, Myanmar (16°32'0.5"N, 97°42'53.5"E): CUMZ 13038 (6 shells; Fig. 2), CUMZ 13039 (15 specimens in ethanol). Pha Boang Cave from Mawlamyine Township, Mawlamyine District, Mon State, Myanmar (16°17'14.04"N, 97°54'4.28"E): CUMZ 13040 (9 adults + 9 juveniles, Fig. 7B), CUMZ 13041 (15 specimens in ethanol).

##### Description.

Shell ovate-fusiform, opaque, and chestnut-brown color; spire blunt. Shell surface nearly smooth or with very thin striations; suture distinct. Whorls 5–6½, convex, regularly growing and bluntly attenuated to apex. Aperture obliquely pear-shaped, basis broader and narrowing towards parietal sinus; peristome detached, thickened, expanded and little reflexed. Superior lamella developed, continuous with spiralis, and low at transition to spiralis. Inferior lamella steeply ascending, distant from superior lamella and ending at peristome. Subcolumellaris emerged, and only visible in oblique view. Principalis running along lateral-dorsal side and anterior end visible through oblique apertural view. Palatal plicae lateral, 7–9: first plica strong and longest; following plicae shorter, parallel, nearly equal in length, gradually reduced towards anterior. Clausilium plate lateral side, slightly broad and rounded at tip.

***Genitalia* (*n* = 5).** Atrium (at) short and slender; penis (p) muscular and cylindrical. Epiphallus (ep) muscular, cylindrical, ca. same length and half of the diameter of penis and broadest at middle part. Penial retractor muscle (pr) attaches proximally to epiphallus. Vas deferens (vd) slender, and shortly bounded at atrium and penis junction (Fig. 8A). Internal sculpture of penis consists of two parts: region near atrium ~ 1/3 of penis length with series of irregular transverse folds, chevron-shaped; region near epiphallus becoming smooth surface and relatively thick with 5–7 longitudinal folds (Fig. 8B). Internal sculpture of epiphallus with elevated papillae arranged in oblique rows (Fig. 8C).

Vagina (v) thick and very short, ~ 1/3 of free oviduct (fo) length. Distal part of pedunculus (dpb) large and long; diverticulum (db) long, ~ 2× length and slightly larger diameter than dpb; proximal part of pedunculus (ppb) slender, ca. equal in length to diverticulum, and bursa copulatrix (bb) with distinct ovate shape (Fig. 8A).

***Radula*.** Each row contains ≥ 39 teeth with half-row formula: central–(lateral)–marginal teeth (1–(11–12)–19+). Central tooth unicuspid with rounded to blunt tip. Lateral teeth bicuspid: endocone large and rounded tip; ectocone very small, pointed tip and located near the base. Marginal teeth asymmetrically tricuspid starting at approximately tooth number 11 or 12: mesocone large and curved to blunt tip; endocone small and located near tip of mesocone; ectocone triangular, pointed tip and located near the base. Outermost teeth have been lost during the preparation process (Fig. 15A).

##### Distribution.

In Myanmar, this species is reported from Kayin, Mon and Tanintharyi (Gude 1914; Szekeres et al. 2021a, b). In addition, the records outside Myanmar are from Kanchanaburi Province, Thailand (Szekeres et al. 2021b), and further in Andaman Islands, but mentioned as dubious record (see Szekeres et al. 2021a: 177). In this survey, three populations were collected from isolated limestone karsts in Salween River Basin with relatively high abundance.

##### Remarks.

*Oospirastoliczkana* can be distinguished from *O.philippiana* by its elongate ovoid shell, pale color, and higher numbers of plicae. In terms of the genital organs, the male internal sculpture of *O.philippiana* has thicker and denser corrugated folds near the atrium than in *O.stoliczkana* (Fig. 10F). Moreover, *O.vespa* can be differentiated from this species by having an acute spire, aperture more oblique and broader, whorls more expanded.

Among the examined specimens, those from the Pha Boang Cave population (24 shells) have larger and darker shells with more widely spaced and up to nine developed palatal plicae. In contrast, specimens from the Kayon Cave (21 shells) and Saddan Cave (15 shells) populations are uniform in terms of a smaller shell and seven closer and weaker plicae (Table 1).

No specimens were found at the type locality ‘Mergui’ [Myeik]; but Szekeres et al. (2021b) reported material from Tanintharyi Region. Instead, multiple populations with high abundance were collected in the vicinity of Salween River Basin in this present study (Fig. 1; Table 1). This agreed with Stoliczka’s (1871: 175) statement that the species was ‘common at the Farm-caves near Moulmein on limestone hills’.

#### ﻿5 Oospira bulbus

Taxon classificationAnimaliaStylommatophoraClausiliidae

(Benson, 1863)

C78E412E-E20D-5646-97D9-C4A55A1627DF

Figs 3C , 7D–F , 8D–F , 18E ; Tables 2 , 3

Clausilia bulbus Benson, 1863: 321. Type locality: “ad ripas fluvii Attaran, non procul ab urbe Moulmein” [banks of Attaran River, Mawlamyine, Mon State, Myanmar]. Pfeiffer 1868: 409. Hanley and Theobald 1870: 12, pl. 24, fig. 5. Sowerby 1875: Clausilia pl. 6, species 51.
Clausilia (Oospira) bulbus –Blanford 1872: 206, pl. 9, fig. 16. Gude 1914: 332.
Clausilia [Phaedusa (Oospira)] bulbus–Boettger 1878: 56.
Oospira (Oospira) bulbus –Nordsieck 2002b: 86. Nordsieck 2007: 23. Preece et al. 2022: 162, fig. 72e.
Oospira bulbus –Szekeres et al. 2021a: 168, fig. 5c, d.

##### Material examined.

Sanbel Cave, Mawlamyine Township, Mon State, Myanmar (16°22'26.0"N, 97°46'22.6"E): CUMZ 13042 (15 shells; 7D–F), CUMZ 13043 (3 specimens in ethanol), JG-C2882 (1 shell).

##### Description.

Shell oblong-ovate, translucent, and brownish; spire rounded and almost flatted to apex. Shell surface with fine and distinct striations; suture wide and distinct. Whorls 5–5½; penultimate whorl rapidly growing and largest; last whorl compressed and narrower. Aperture obliquely pear-shaped, basis rounded and narrowing towards parietal sinus; peristome detached, thickened, slightly expanded, and not reflexed. Superior lamella sharp, oblique, continuous with spiralis, and low at transition to spiralis. Inferior lamella steeply ascending, distant from superior lamella and ending at peristome. Subcolumellaris emerged and invisible in oblique view. Principalis running along lateral-dorsal side and anterior end visible through oblique apertural view. Palatal plicae lateral, five: first plica longest; following four plicae parallel, equal in length and spacing, and slightly reduced towards anterior. Clausilium plate lateral side and narrow.

***Genitalia*** (*n* = 2). Atrium (at) short and slender; penis (p) muscular and cylindrical. Epiphallus (ep) muscular, cylindrical, ca. same length and half diameter of penis, and broadest at middle part. Penial retractor muscle (pr) attaches proximally to epiphallus. Vas deferens (vd) slender, and shortly bounded at atrium and penis junction (Fig. 8D). Internal sculpture of penis consists of two parts: region near atrium and ~ 1/2 of penis length with series of irregular transverse folds, chevron-shaped; region near epiphallus becoming smooth, with 5–7 slightly wavy longitudinal folds (Fig. 8E). Internal sculpture of epiphallus with elevated papillae arranged in oblique rows (Fig. 8F).

Vagina (v) thick and short, ~ 1/2 of free oviduct (fo) length. Distal part of pedunculus (dpb) large and long; diverticulum (db) long, ~ 2× in length and slightly smaller in diameter than dpb; proximal part of pedunculus (ppb) slender, ca. equal in length to diverticulum and bursa copulatrix (bb) with distinct ovate shape (Fig. 8D).

##### Distribution.

*Oospirabulbus* has only been reported from Myanmar and is possibly endemic in the limestone hills along the lower course of Attaran River. The specimens examined herein were collected near the type locality.

##### Remarks.

*Oospiraovata* can be separated from this species by its a smoother striated shell, attenuated spire and usually seven plicae. In terms of the genital organs, the male internal sculpture of *O.ovata* has longer V-shaped folds in the region near the atrium and rounded papillae in epiphallus than this species. Although *O.bulbus* has a similar shell form to *Atractophaeduspyknosoma* (Gittenberger & Vermeulen, 2001) from Vietnam, *A.pyknosoma* has a narrower spire, visible subcolumellaris in frontal view, peristome reflected with nearly double lip and first palatal plicae shorter than the following plicae (see Gittenberger and Vermeulen 2001: figs 1–10).

#### ﻿6 Oospira ovata

Taxon classificationAnimaliaStylommatophoraClausiliidae

(Blanford, 1872)

47FFDD4A-A622-59CE-B1D0-3143D01CCD53

**Figure 9.**
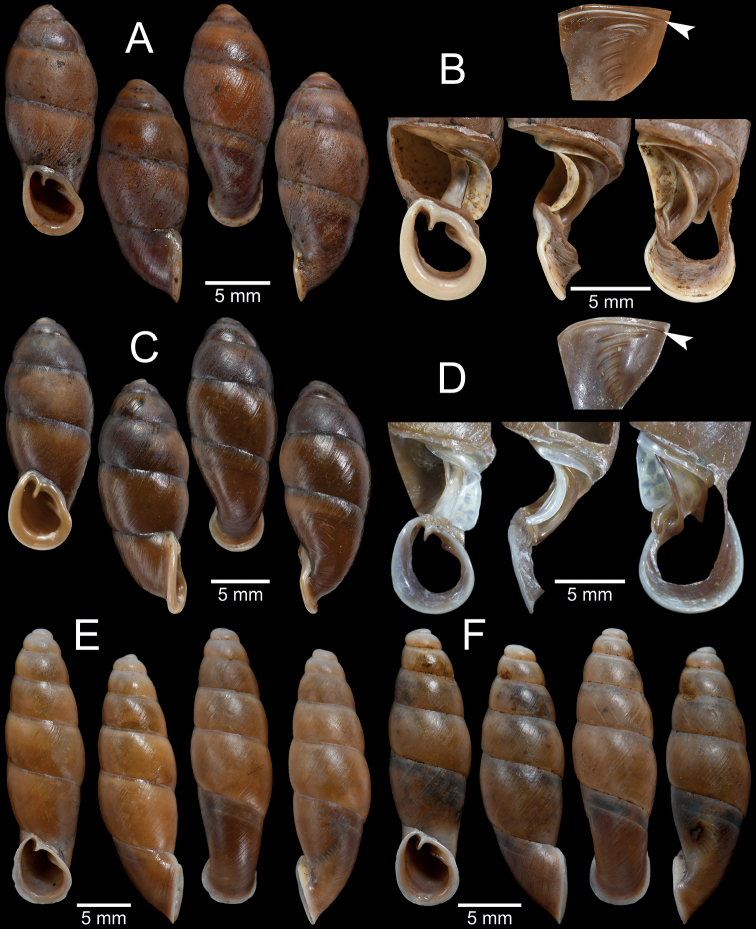
Shell and clausilial apparatus of **A–C***Oospiraovata*, specimen CUMZ 13044 from Pathein Mountain, Mon State **A, C** shells **B** clausilial apparatus and **D–F***Oospirastoliczkana*, specimen CUMZ 13046 from Phra Cave, Tanintharyi Region **D** clausilial apparatus **E, F** shells. White arrows indicate principalis.

**Figure 10.**
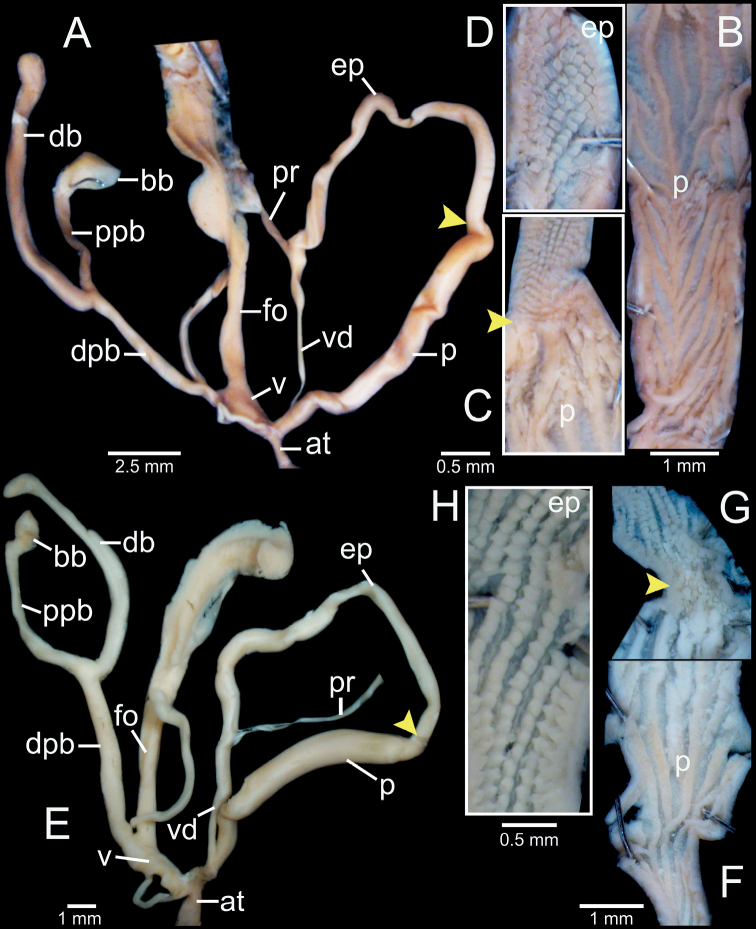
Genital anatomy of **A–D***Oospiraovata*, specimen CUMZ 13045 **A** whole reproductive system **B** internal sculpture of penis **C** transition from penis to epiphallus **D** internal sculpture of epiphallus and **E–H***Oospirastoliczkana*, specimen CUMZ 13047 **E** whole reproductive system **F** internal sculpture of penis **G** transition from penis to epiphallus **H** internal sculpture of epiphallus. Yellow arrows indicate approximate transitional position from penis to epiphallus.

Figs 3D , 9A–C , 10A–D , 15B , 19G ; Tables 2 , 3

Clausilia (Oospira) ovata Blanford, 1872: 206, pl. 9, fig. 17. Type locality: “Ad Nattoung, juxta ripam Attaran fluminis, haud procul a Moulmain in Barma” [Shwe Nat Taung, Mawlamyine, Mon State, Myanmar]. Nevill 1878: 183. Gude 1914: 334.
Clausilia ovata –Hanley and Theobald 1874: 48, pl. 118, fig. 4. Sowerby 1875: Clausilia pl. 7, species 60. Pfeiffer 1877: 469.
Clausilia [Phaedusa (Oospira)] ovata–Boettger 1878: 56.
Oospira (Oospira) ovata –Nordsieck 2002b: 86. Nordsieck 2007: 24.
Oospira ovata –Szekeres et al. 2021a: 175, 176, fig. 8a, b.

##### Material examined.

Pathein Mountain, Mawlamyine Township, Mon State, Myanmar (16°14'7.5"N, 97°56'48.1"E): CUMZ 13044 (25 shells, Fig. 9A–C), CUMZ 13045 (1 specimen in ethanol), JG-C2883 (2 shells).

##### Description.

Shell ovate or pupiform, opaque and chestnut-brown; spire blunt. Shell surface nearly smooth or with very thin growth lines; suture distinct. Whorls 5–5½ convex, rapidly growing; penultimate and antepenultimate whorls equally broad and rounded to apex. Aperture obliquely pear-shaped, basis rounded and narrowing towards parietal sinus; peristome detached, thickened, expanded and little reflexed. Superior lamella well developed, continuous with spiralis and low at transition to spiralis. Inferior lamella steeply ascending, distant from superior lamella and ending at peristome. Subcolumellaris emerged and only visible in oblique view. Principalis running along lateral-dorsal side and anterior end visible through oblique apertural view. Palatal plicae lateral, six or seven: first plica strong and longest; following plicae short, parallel, equal in length and gradually reduced towards anterior. Clausilium plate lateral side and narrow.

***Genitalia*** (*n* = 1). Atrium (at) short and slender; penis (p) muscular and cylindrical. Epiphallus (ep) muscular, cylindrical, ~ 1.5× diameter of penis, and broadest close to penis. Penial retractor muscle (pr) attaches proximally to epiphallus. Vas deferens (vd) slender and shortly bounded at atrium and penis junction (Fig. 10A). Internal sculpture of penis consists of two parts: region near atrium, ~ 2/3 of penis length with moderately thick transverse folds arranged in V-shaped rows; region near epiphallus with smooth surface and four or five longitudinal folds (Fig. 10B, C). Internal sculpture of epiphallus with rounded papillae arranged in oblique rows (Fig. 10D).

Vagina (v) thick, short, and almost half of free oviduct (fo) length. Distal part of pedunculus (dpb) large and long; diverticulum (db) large, slightly longer, and larger in diameter than dpb; proximal part of pedunculus (ppb) slender, ~ 2/3 diverticulum length and bursa copulatrix (bb) with distinct ovate shape (Fig. 10A).

***Radula*.** Each row contains ~ 61 teeth with half-row formula: central–(lateral)–marginal teeth (1–(12–13)–26). Central tooth unicuspid with dull tip. Lateral teeth bicuspid; endocone large with curved to dull tip; ectocone very tiny to small and pointed tip. Marginal teeth asymmetrically tricuspid starting at approximately tooth number 12 or 13: mesocone large and dull tip; ectocone and endocone small with pointed tips. Outer marginal teeth increasingly asymmetrical and shorter than inner marginal teeth (Fig. 15B).

##### Distribution.

At present, *O.ovata* is only known from Myanmar. Many shells were collected in this study; it is likely endemic to the valley of Attaran River.

##### Remarks.

*Oospiraovata* and *O.philippiana* are generally similar in shell morphology, but *O.ovata* has a more expanded spire. The male internal sculpture of *O.ovata* has longer V-shaped folds in the region near the atrium and rounded papillae pattern in epiphallus, whereas *O.philippiana* has shorter V-shaped folds and more elevated and pointed papillae in epiphallus.

#### ﻿7 Oospira stoliczkana

Taxon classificationAnimaliaStylommatophoraClausiliidae

(Sykes, 1893)

F527508A-FC70-5856-A0B1-60147449CD7B

Figs 9D–F , 10E–H , 19L ; Tables 2 , 3

Clausilia vespa –Stoliczka 1872: 209, pl. 9, fig. 15a. (non Gould, 1856)
Clausilia vespa –Pfeiffer 1877: 468 (in part).
Clausilia (Oospira) stoliczkana Sykes, 1893: 166. Type locality: “Tavoy, Burmah” [Dawei, Tanintharyi Region, Myanmar]. Gude 1914: 335.
Oospira (Oospira) stoliczkana –Nordsieck 2002b: 86. Nordsieck 2007: 24.
Oospira stoliczkana –Szekeres et al. 2021a: 178, 179, fig. 9a.

##### Material examined.

Phra Cave, Tanintharyi Region, Myanmar (11°13'46.2"N, 99°10'34.3"E): CUMZ 13046 (4 shells; Fig. 9D–F), CUMZ 13047 (1 specimen in ethanol).

##### Description.

Shell elongate ovoidal, glossy, translucent, and horny brown; spire blunt. Shell surface with very thin striations to smooth surface; suture wide and distinct. Whorls 6–6½, convex, regularly growing and bluntly attenuated to apex. Aperture obliquely pear-shaped, basis rounded and narrowing towards parietal sinus; peristome detached, thin and little expanded. Superior lamella developed, sharp, continuous with spiralis and low at transition to spiralis. Inferior lamella steeply ascending, distant from superior lamella and ending at peristome. Subcolumellaris emerged and only visible in oblique view. Principalis running along lateral-dorsal side and anterior end visible through oblique apertural view. Palatal plicae lateral, 8–10: first plica strong and longest; following four plicae parallel and equal in length, and remaining plicae shorter, closer, gradually reduced towards anterior. Clausilium plate lateral side and slightly broad at tip.

***Genitalia*** (*n* = 1). Atrium (at) short and slender; penis (p) muscular and cylindrical. Epiphallus (ep) muscular, cylindrical, slightly longer by 1.5× diameter of penis, and broadest at proximal part. Penial retractor muscle (pr) attaches proximally to epiphallus. Vas deferens (vd) slender, and shortly bounded at atrium and penis junction (Fig. 10E). Internal sculpture of penis consists of two parts: region near atrium, ~ 1/4 length of penis with irregular transverse fleshy folds arranged in nearly V-shaped rows (Fig. 10F); region near epiphallus becoming smooth with oblique longitudinal folds (Fig. 10G). Internal sculpture of epiphallus with elevated papillae arranged in nearly oblique rows (Fig. 10H).

Vagina (v) large, short, and almost half the length of free oviduct (fo). Distal part of pedunculus (dpb) large and long; diverticulum (db) long, ca. same length as dpb; proximal part of pedunculus (ppb) slender, almost equal in length to diverticulum, and bursa copulatrix (bb) small, ovateshape (Fig. 10E).

##### Distribution.

This species is known from Mon State, along with its type locality in Tanintharyi Region; no reports have been made outside Myanmar to date (Szekeres et al. 2021a).

##### Remarks.

*Oospirastoliczkana* can be distinguished from all other species with an ovate-fusiform shell (*O.philippiana*, *O.ovata*, *O.bulbus*, and *O.vespa*) by its elongate ovoidal shell, thinner peristome, and 8 to 10 palatal plicae. Although only one ethanol-preserved specimen could be examined, the internal sculpture of the penis and epiphallus of *O.stoliczkana* differs from *O.philippiana*, *O.bulbus*, and *O.ovata* by its very short V-shaped folds in the region near the atrium and more elevated and pointed papillae in epiphallus.

Some of the examined specimens had a thin and translucent shell, with more distinct palatal plicae.

###### ﻿*Oospira* species group with long and slender fusiform shell

In Myanmar, this group comprises 15 species including two new ones. Six of these species are examined herein; the remaining nine are *O.arakana* (Stoliczka, 1872), *O.decollata* (Likharev, 1962), *O.fusiformis* (Blanford, 1865), *O.gracilior* (Hanley & Theobald, 1870), *O.insignis* (Gould, 1843), *O.limborgi* Grego & Szekeres, 2021, *O.malaisei* Nordsieck, 1973, *O.mongmitensis* Grego & Szekeres, 2021 and *O.sardicola* Grego & Szekeres, 2021.

#### ﻿8 Oospira gouldiana

Taxon classificationAnimaliaStylommatophoraClausiliidae

(Pfeiffer, 1857)

B4A54E44-B7C5-5B83-A0F5-81B4A233884F

**Figure 11.**
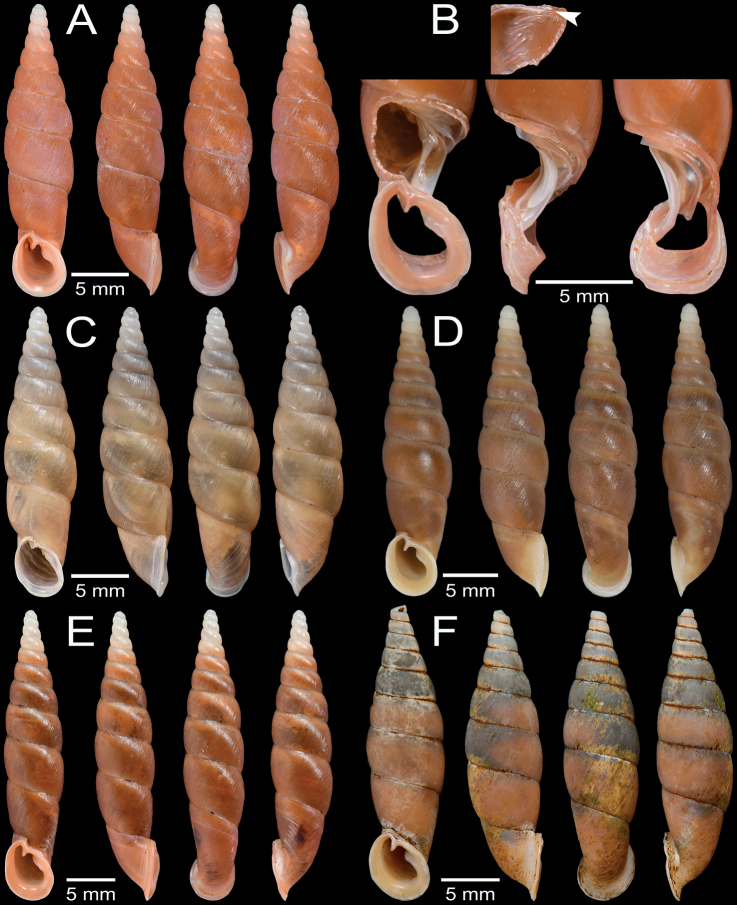
Shell and clausilial apparatus of *Oospiragouldiana***A–C** specimen CUMZ 13048 from Bardai Mountain, Kayin State **A** typical pinkish shell form **B** clausilial apparatus **C** not fully adult shell **D** yellowish shell form, specimen CUMZ 13049 from Kaw Ka Thaung Cave, Kayin State **E** slender shell form, specimen CUMZ 13052 from Taung Lay Cave, Kayin State **F** ovate shell form, specimen CUMZ 13053 from Waiponla Mountain, Kayin State. White arrow indicates principalis.

Figs 3E , 11 , 13A–D , 15C , 18J ; Tables 2 , 3

Clausilia gouldiana Pfeiffer, 1857: 259. Type locality: “Mergui imperii Birmani” [Myeik Islands, Tanintharyi Region, Myanmar]. Pfeiffer 1860: 123, pl. 34, figs 18–20. Pfeiffer 1868: 409. Stoliczka 1872: 208. Hanley and Theobald 1874: 48, pl. 118, figs 2, 3. Sowerby 1875: Clausilia pl. 16, species 148.
Clausilia (Phaedusa) gouldiana –Blanford 1872: 203, pl. 9, fig. 10.
Clausilia [Phaedusa (Pseudonenia)] gouldiana–Boettger 1878: 54. Pfeiffer and Clessin 1881: 391. Gude 1914: 314–316, fig. 108.
Oospira (Oospira) gouldiana –Nordsieck 2002b: 86. Nordsieck 2007: 23.
Oospira gouldiana –Szekeres et al. 2021a: 171, fig. 6d–f. Szekeres et al. 2021b: 41.

##### Materials examined.

Bardai Mountain, Hpa-an Township, Hpa-an District, Kayin State, Myanmar (16°59'10.4"N, 97°42'19.8"E): CUMZ 13048 (25 specimens in ethanol, Fig. 11A–C). Kaw Ka Thaung Cave, Hpa-an Township, Hpa-an District, Kayin State, Myanmar (16°49'40.4"N, 97°42'31.0"E): CUMZ 13049 (23 specimens in ethanol, Fig. 11D). Lun Nga Mountain, Hpa-an Township, Hpa-an District, Kayin State, Myanmar (16°44'53.2"N, 97°47'09.5"E): CUMZ 13050 (18 specimen in ethanol). Sadhdan Cave, Hpa-an Township, Hpa-District, Kayin State, Myanmar (16°44'23.4"N, 97°43'04.2"E): CUMZ 13051 (2 incomplete shells). Taung Lay Cave, Hpa-an Township, Hpa-an District, Kayin State, Myanmar (17°11'40.3"N, 97°37'47.0"E): CUMZ 13052 (30 shells; Fig. 11E). Waiponla Mountain, Hpa-an Township, Hpa-an District, Kayin State, Myanmar (16°56'7.4"N, 97°42'56.8"E): CUMZ 13053 (2 incomplete shells, Fig. 11F), CUMZ 13054 (2 specimens in ethanol, lost apical whorls). Rathye Pyan Cave, Hpa-an Twonship, Hpa-an District, Kayin State, Myanmar (16°50'6.2"N, 97°34'14.5"E): CUMZ 13055 (1 incomplete shell).

##### Description.

Shell fusiform, translucent, brownish to reddish pink, and 3–4 white apical whorls; spire regularly attenuated. Shell surface finely striated to nearly smooth surface; suture impressed and distinct. Whorls 10–12 convex, regularly growing or sometimes rapidly growing after antepenultimate whorl and attenuated to apex. Aperture pear-shaped, basis broader and narrowing towards parietal sinus; peristome slightly protruded, thickened, expanded, and indistinctly doubled. Superior lamella developed, continuous with spiralis and slightly low at transition to spiralis. Inferior lamella steeply ascending, distant from superior lamella and ending at peristome. Subcolumellaris emerged and invisible in oblique view. Principalis running along lateral-dorsal side and anterior end visible through oblique apertural view. Palatal plicae lateral, five or six: first plica strong and longest; following plicae short, more or less parallel; fourth or fifth plica longer than middle plicae. Clausilium plate lateral side and narrow.

***Genitalia*** (*n* = 5). Atrium (at) short and slender; penis (p) long, slender, and shortly narrower at transition to epiphallus. Epiphallus (ep) slender, ca. same length as penis, slightly smaller diameter, and broadest at middle. Penial retractor muscle (pr) attaches proximally to epiphallus. Vas deferens (vd) thin and shortly bounded at penis (Fig. 13A). Internal sculpture of penis with smooth surfaces and five or six thin longitudinal folds (Fig. 13B). Internal sculpture of epiphallus with irregular corrugated pattern (Fig. 13D) and sometimes with distinct irregular transverse fold and papillae arranged in oblique rows.

Vagina (v) thick, cylindrical, and almost half of free oviduct (fo) length. Distal part of pedunculus (dpb) large and middle part broadest; diverticulum (db) slender, very long, ca. same length as distal part and proximal part of pedunculus; proximal part of pedunculus (ppb) slender, nearly same length as (dpb), and bursa copulatrix (bb) ovoid (Fig. 13A).

***Radula*.** Each row contains ≥ 39 teeth with half-row formula: central–(lateral)–marginal teeth (1–(11–12)–19). Central tooth tricuspid with large and triangular mesocone, and ectocones very small and pointed tips. Lateral teeth bicuspid: endocone large and triangular; ectocone very small and pointed tip. Marginal teeth asymmetrically tricuspid starting at approximately tooth number 11 or 12: endocone small located near the tip; mesocone large and dull to blunt tip; ectocone with triangular shape, pointed tip and located near the base (Fig. 15C).

##### Distribution.

*Oospiragouldiana* is the most abundant species of the genus in Myanmar, occurring mainly in the south-eastern part of the country. This species was abundant at seven limestone outcrops in Kayin State (Fig. 1, Table 1). It was recorded from Bago, Kayin, Mon, and Tanintharyi in Myanmar, and the distribution was later expanded to include parts of Thailand (Gude 1914; Szekeres et al. 2021a, b).

##### Remarks.

*Oospiragouldiana* specimens from Taung Lay Cave are quite similar to *O.malaisei* from Kachin State in terms of shell form. But *O.malaisei* (Fig. 17C) shows a more vertical and broader aperture, reflected peristome, closely and rapidly attenuated apical whorls, and inferior lamella more straight ascending at peristome. In contrast, Taung Lay Cave specimens have an oblique and rounded aperture, less reflected peristome, regularly attenuated whitish apical whorls and inferior lamella more spirally ending at peristome. Anatomically, *O.malaisei* possesses an epiphallus of ~ 1/2 the penis length, while in *O.gouldiana* epiphallus and penis have almost equal lengths (see Nordsieck 1973: fig. 26).

*Oospiragouldiana* also shares a similar shell morphology with *O.insignis* exhibiting a regularly attenuated spire with whitish apical whorls, palatal plicae five, and a broad pear-shaped aperture. Nevertheless, *O.gouldiana* has a more attenuated spire, more parallel palatal plicae, aperture more vertical, and less glossy than *O.insignis*. In addition, this species can easily be separated from *O.shanensis* by its pale color, shorter and oblique palatal plicae, inferior lamella more spirally and closely ascending from spiralis and superior lamella. *Oospirapenangensis* differs from this species by its less ventricose, narrower, and more vertical subquadrate aperture, inferior lamella ending straight at peristome and weaker superior lamella from apertural view.

*Oospiragouldiana* is highly variable in terms of shell color and shape. The typical form was found in the Bardai and Kaw Ka Thaung populations, showing a yellowish to pinkish color with a regular fusiform shape (Fig. 11A, D), while the Taung Lay population have a slenderer shell and the Waiponla population have a more bulging shell (Fig. 11E, F). In addition, the arrangement of palatal plicae shows much variation among the populations. For instance, the Bardai population has irregularly or obliquely arranged plicae (nearly vertical), Kaw Ka Thaung population has parallelly arranged plicae, while the Waiponla and Lun Nga populations have very weak and short parallelly arranged palatal plicae. Furthermore, a very short plica (dot-like) located near the anteriormost palatal plicae next to subcolumellaris is also observed in the Bardai population.

Except for the Sadhdan, Taung Lay, and Rathye Pyan populations, for which genitalia could not be examined, the remaining populations are similar in external genital morphology, with only slightly differing thickness of the longitudinal folds of the internal sculpture of the penis being observed. However, these four populations inhabit a very small geographical area, hence we attribute this minor difference to morphological variation.

#### ﻿9 Oospira andersoniana

Taxon classificationAnimaliaStylommatophoraClausiliidae

(Möllendorff, 1882)

E4CE4904-54E2-5D51-8005-20BBDFA278C4

**Figure 12.**
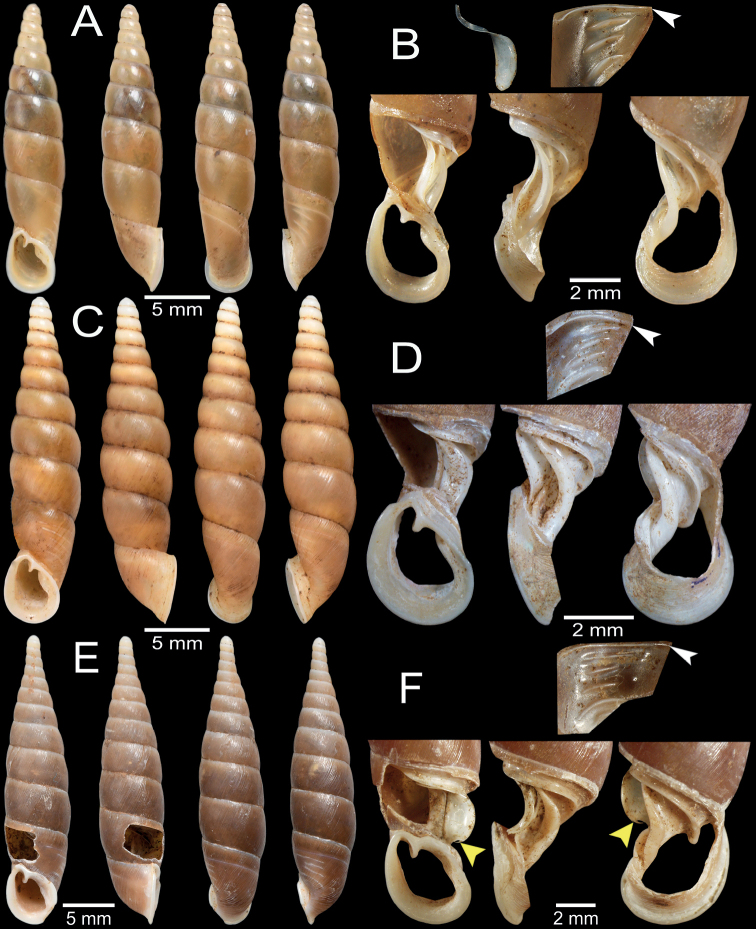
Shell and clausilial apparatus of **A, B***Oospiraandersoniana*, specimen CUMZ 13056 from Phra Cave, Tanintharyi Region **A** shell **B** clausilial apparatus **C, D***Oospiramagna*, specimen CUMZ 13058 from Bayin Nyi Cave, Kayin State **C** shell **D** clausilial apparatus and **E, F***Oospirashanensis*, specimen CUMZ 13060 from Nanthe Cave, Shan State **E** shell **F** clausilial apparatus. White arrows indicate principalis and yellow arrows indicate clausilium hook.

**Figure 13.**
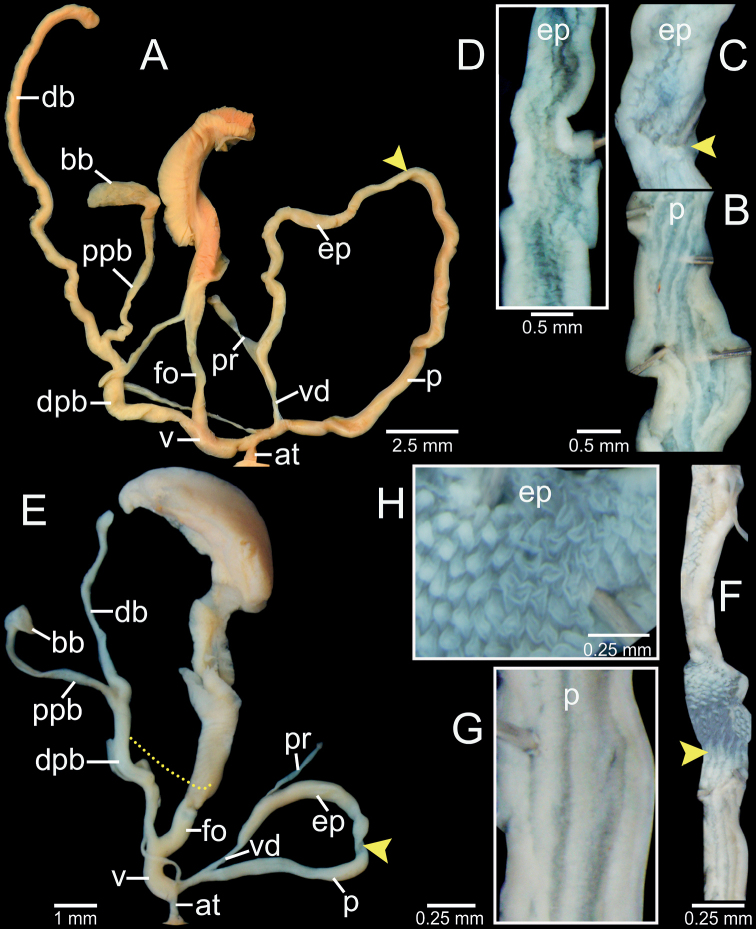
Genital anatomy of **A–D***Oospiragouldiana*, specimen CUMZ 13049 **A** whole reproductive system **B** internal sculpture of penis **C** transition from penis to epiphallus **D** internal sculpture of epiphallus and **E–H***Oospiraandersoniana*, specimen CUMZ 13057 **E** whole reproductive system **F** overview of internal sculpture of penis and epiphallus **G** internal sculpture of penis **H** internal sculpture of epiphallus. Yellow arrows indicate approximate transitional position from penis to epiphallus.

Figs 12A, B , 13E–H , 15D , 18B ; Tables 2 , 3

Clausilia (Pseudonenia) andersoniana Möllendorff, 1882: 12, pl. 1, fig. 13. Type locality: “In insula Mergui provincise Tenasserim” [Myeik Islands, Tanintharyi Region, Myanmar].
Clausilia [Phaedusa (Pseudonenia)] andersoniana–Gude 1914: 317.

##### Material examined.

The type specimens could not be located in the Senckenberg Museum, Frankfurt (K.-O. Nagel and S. Hof, pers. comm., April 2022). Phra Cave, Tanintharyi Region, Myanmar (11°13'46.2"N, 99°10'34.3"E): CUMZ 13056 (9 shells; Fig. 12A, B), CUMZ 13057 (25 specimens in ethanol), JG-C2880 (2 shells).

##### Description.

Shell fusiform, translucent, and pale yellowish brown color; spire regularly attenuated. Shell surface glossy and almost smooth surface; suture impressed and distinct. Whorls 9–10, little convex, regularly growing and attenuated to apex. Aperture pear-shaped, and basis broader and narrowing towards parietal sinus; peristome protruded, little thickened and expanded. Superior lamella developed, continuous with spiralis, and indistinct at transition to spiralis. Inferior lamella steeply ascending, distant from superior lamella and ending at peristome. Subcolumellaris emerged and invisible in oblique view. Principalis running along lateral-dorsal side and anterior end visible through oblique apertural view. Palatal plicae lateral, 3–5 (usually 3): all plicae very strong, parallel, equal length, spacing, and one or two small plicae sometimes present in middle and at lowest. Clausilium plate lateral side and narrow.

***Genitalia*** (*n* = 5). Atrium (at) short and slender; penis (p) almost cylindrical and shortly narrower at transition to epiphallus. Epiphallus (ep) cylindrical, ca. same length and diameter as penis. Penial retractor muscle (pr) attaches proximally to epiphallus. Vas deferens (vd) thin and shortly bounded at penis (Fig. 13E). Internal sculpture of penis with smooth surfaces and with relatively thickened four or five longitudinal folds (Fig. 13F, G). Internal sculpture of epiphallus with large and scattered papillae arranged obliquely to irregularly spaced rows (Fig. 13H).

Vagina (v) thick, cylindrical, and almost equal to free oviduct (fo) length. Distal part of pedunculus (dpb) large and long; diverticulum (db) thin and ca. same length as dpb; proximal part of pedunculus (ppb) slender, nearly equal in length to diverticulum, and bursa copulatrix (bb) small, ovoid (Fig. 13E).

***Radula*.** Each row contains ~ 45 teeth with half-row formula: central–(lateral)–marginal teeth (1–(10–11)–22). Central tooth tricuspid: mesocone triangular with dull cusp; ectocones small and pointed tip. Lateral teeth bicuspid: endocone large and with dull cusp; ectocone small with tip pointed laterally. Marginal teeth asymmetrically tricuspid starting at approximately tooth number 10 or 11: endocone very tiny; mesocone large, elongate, blunt tip; ectocone small, triangular, pointed tip. Outermost becoming smaller and more asymmetrical than inner teeth (Fig. 15D).

##### Distribution.

Currently *O.andersoniana* is only known from Tanintharyi Region, Myanmar.

##### Remarks.

This species was described based on two shells collected by the Scottish zoologist, John Anderson (1833–1900), approximately from southern Myanmar, and an illustration was included in the original publication (Möllendorff 1882; Alcock 1902). Then, it was re-described and suggested to be very similar to *O.gouldiana* by Gude (1914). Despite the type specimen being mentioned in the type catalogue of the Clausiliidae in the Naturmuseum Senckenberg by Zilch (1954), this nominal species seems mysterious and is, therefore, listed as uncertain (taxon inquirendum) in MolluscaBase (2022). The type specimens of this species are presumably lost. However, Möllendorff (1882) provided a very detailed description and illustration of the species. Our newly collected specimens from the Tanintharyi Region have a thinly striated shell, smooth surface, thin peristome, mostly nine whorls and three palatal plicae, which matches well with the diagnostic characteristics of this species.

In this survey, *Oospiraandersoniana* and *O.stoliczkana* were collected from the same locality in the Tanintharyi Region. These two species are similar in possessing a nearly smooth shell glossy surface, thin peristome, pale yellowish color, and less developed inferior lamella. However, *O.stoliczkana* exhibits a shorter, blunt spire, broader clausilium and several palatal plicae (from 8 to 10).

Compared with the other congeners, *Oospiraandersoniana* can be distinguished from *O.insignis*, *O.gouldiana*, and *O.magna* by its less ventricose shell, smoother surface and pale color, peristome and inferior lamella weaker, and palatal plicae longer and more uniformly arranged. In contrast, *O.insignis* possesses oblique palatal plicae, more ventricose and darker colored shell, while *O.gouldiana* has a ventricose shell, spire more attenuated and with whitish apical whorls, inferior lamella more bent, aperture thicker and broader, and palatal plicae closer and more oblique. In addition, *O.magna* can be distinguished from *O.andersoniana* by having a finely and densely striated shell surface, inferior lamella well developed, clausilium in ventral position, and palatal plicae longer and more ventral (nearly horizontal). In terms of the genitalia, *O.andersoniana* has a short male organ, internal penis with large longitudinal folds and epiphallus with a distinct reticulated papillae pattern. In contrast, *O.gouldiana* and *O.magna* possess slender and longer male organs, internal penis with thinner and denser longitudinal folds, and epiphallus with irregularly arranged reticulated papillae patterns. Furthermore, *O.limborgi* Grego & Szekeres, 2021, also described from the Tanintharyi Region, shares some characteristics with this species in having the same number of palatal plicae, nearly triangular aperture, glossy and pale yellowish color (see Szekeres et al. 2021a: fig. 7c).

Shell variation was observed with respect to a small and short plica between the first and second palatal plicae.

#### ﻿10 Oospira magna

Taxon classificationAnimaliaStylommatophoraClausiliidae

(Gude, 1914)

43FFA404-8EAB-5308-8031-AC33653DA815

**Figure 14.**
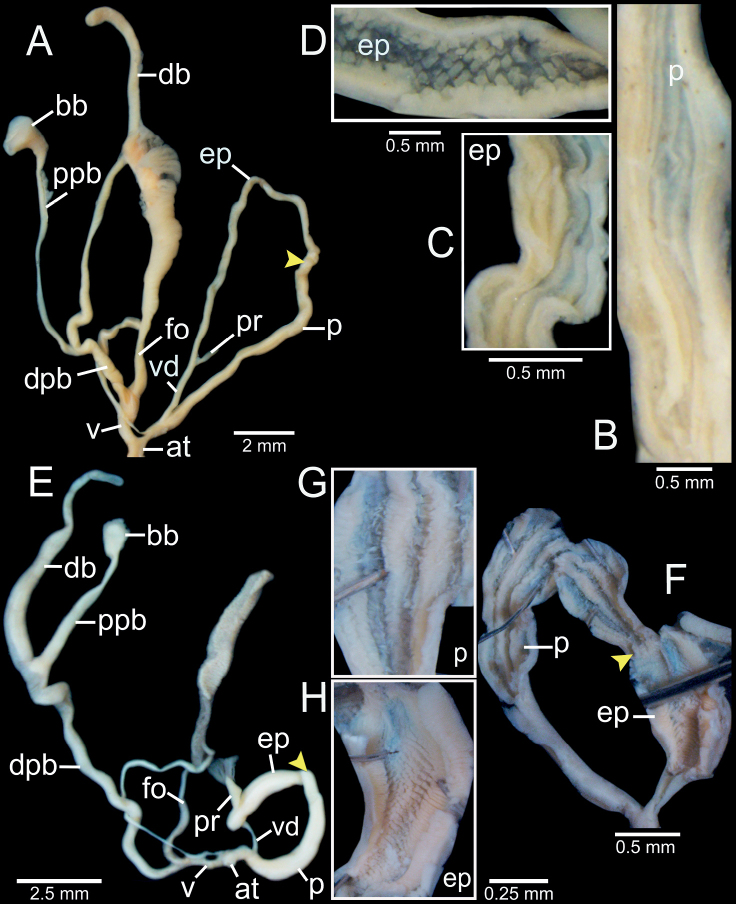
Genital anatomy of **A–D***Oospiramagna*, specimen CUMZ 13059 **A** whole reproductive system **B** internal sculpture of penis near atrium **C** internal sculpture of penis near epiphallus **D** internal sculpture of epiphallus and **E–H***Oospirashanensis*, specimen CUMZ 13061 **E** whole reproductive system **F** overview internal sculpture of penis and epiphallus **G** internal sculpture of penis **H** internal sculpture of epiphallus. Yellow arrows indicate approximate transitional position from penis to epiphallus.

Figs 12C, D , 14A–D , 19B ; Tables 2 , 3

Clausilia [Phaedusa (Pseudonenia)] gouldianavar.magna Gude, 1914: 316, fig. 109. Type locality: “Moulmain and Phaboo” [Mawlamyine, Mon State and Pabu, Hpa-an, Kayin State, Myanmar].

##### Materials examined.

Bayin Nyi Cave, Hpa-an Township, Kayin State, Myanmar (16°58'10.1"N, 97°29'30.6"E): CUMZ 13058 (3 shells + 6 incomplete shells, Fig. 12C, D), CUMZ 13059 (1 specimen in ethanol).

##### Description.

Shell fusiform, translucent, yellowish brown and 3–4 white apical whorls; spire regularly attenuated. Shell surface nearly smooth to with thin growth lines; suture deep and distinct. Whorls 10–11, convex, regularly growing and attenuated to apex. Aperture oval-pear-shaped, less oblique, basis broader and narrowing towards parietal sinus; peristome protruded, thickened, and slightly expanded. Superior lamella developed, continuous with spiralis, and indistinct at transition to spiralis. Inferior lamella well developed, steeply ascending, distant from superior lamella and ending at peristome. Subcolumellaris emerged and invisible in oblique view. Principalis running along ventral-lateral side and anterior end visible through oblique apertural view. Palatal plicae ventral-lateral, 4–6 (usually 4): first plica strong and longest; second and third plicae short, parallel, oblique, and equal in length; fourth plica longer and stronger than middle plicae. Clausilium plate ventral side and narrow.

***Genitalia*** (*n* = 1). Atrium (at) short and relatively large; penis (p) almost cylindrical and shortly narrower at transition to epiphallus. Epiphallus (ep) slender, ca. same length as penis and smaller diameter. Penial retractor muscle (pr) attaches proximally to epiphallus. Vas deferens (vd) almost same diameter as epiphallus and shortly bounded at penis (Fig. 14A). Internal sculpture of penis with smooth surface and ca. four or five thin longitudinal rows (Fig. 14B), and folds slightly enlarged near epiphallus (Fig. 14C). Internal sculpture of epiphallus with large papillae arranged in oblique rows (Fig. 14D).

Vagina (v) thick, cylindrical, and almost equal in length to free oviduct (fo). Distal part of pedunculus (dpb) cylindrical and long; diverticulum (db) very long, slightly enlarged at basis, then gradually slenderer, ~ 3× times length of dpb; proximal part of pedunculus (ppb) slender, slightly short, ~ 3/4 diverticulum length; bursa copulatrix (bb) large, ovoid (Fig. 14A).

##### Distribution.

*Oospiramagna* is only recorded from the southern part of the country to the limestone area of the Salween River Basin in Mon and Kayin States, Myanmar.

##### Remarks.

Originally, *Oospiramagna* was proposed as a subspecies of *O.gouldiana* based on its distinct less ventricose shell and seven palatal plicae (Gude 1914). Later Szekeres et al. (2021b) recognized this as a morphological variation within the variable species, *O.gouldiana*. From our recent field survey, several specimens were collected, and their shell morphology matched well with the diagnostic characters of *O.magna*. Although *O.magna* is highly similar to *O.gouldiana* in shell form, it can be distinguished mainly by having a less ventricose shell, almost uniform and ventrally longer palatal plicae, more developed inferior spiral lamella, aperture less oblique, last whorl more compressed, spiralis starting deeply ventral, and clausilium ventrally seated. In genitalia, *O.gouldiana* exhibits a long distal part of pedunculus almost the same length as the proximal part of pedunculus and an internal sculpture of epiphallus with a thin and irregular corrugated pattern. These differences in the shell and genitalia characters are greater than the intraspecific variation and those characters were not observed as a morphological variation among the *O.gouldiana* populations examined; therefore, we have raised *O.magna* to a distinct species. *Oospirazediopsis* sp. nov. differ from *O.magna* by its a more ventricose shell with a smoother surface, apical whorl rapidly attenuated, dark reddish color, clausilium and palatal plicae laterally seated, and inferior lamella straight ascending.

#### ﻿11 Oospira shanensis

Taxon classificationAnimaliaStylommatophoraClausiliidae

Grego & Szekeres, 2021

A38CC937-BCE4-5D36-AC5F-23582B3618DE

**Figure 15.**
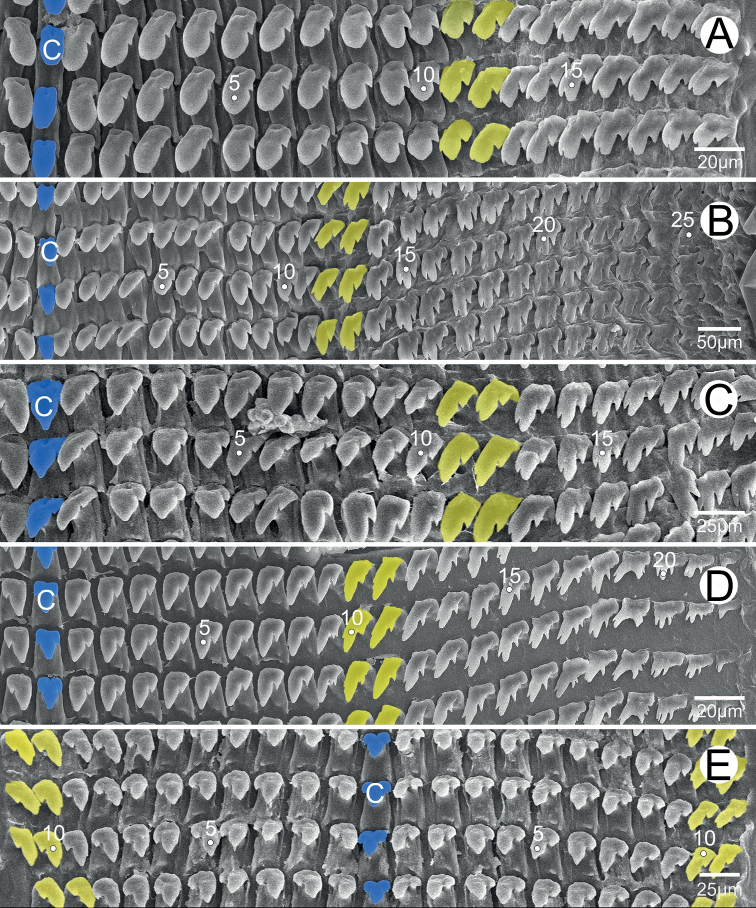
Radula morphology **A***Oospiraphilippiana*, specimen CUMZ 13039 **B***Oospiraovata*, specimen CUMZ 13044 **C***Oospiragouldiana*, specimen CUMZ 13049 **D***Oospiraandersoniana*CUMZ 13057 **E***Oospirashanensis*, specimen CUMZ 13061. Blue color indicates central teeth row; yellow color indicates the transition from lateral to marginal teeth. ‘C’ indicates central tooth.

Figs 3F , 12E, F , 14E–H , 15E , 19J ; Tables 2 , 3

Oospira shanensis Grego & Szekeres in Szekeres et al. 2021a: 177, 178, fig. 8e, f. Type locality: “Shan” [Shan State, Myanmar].

##### Material examined.

Limestone outcrop at Nanthe Cave, Kalaw City, Shan State, Myanmar 20°35'16.6"N, 96°37'57.2"E: CUMZ 13060 (5 shells, Fig. 12E, F), CUMZ 13061 (4 specimens in ethanol).

##### Description.

Shell fusiform, opaque, and dark yellowish color; 5–6 apical whorls rapidly attenuated. Shell surface glossy and almost smooth; suture impressed and distinct. Whorls 12½–13, last four whorls regularly growing and then rapidly attenuated to apex. Aperture obliquely or elliptical, basis oblique and broadest and narrowing towards parietal sinus; peristome attached, thickened, and expanded. Superior lamella developed, continuous with spiralis and indistinct at transition to spiralis. Inferior lamella steeply ascending, anterior end weakly visible in apertural view, very distant from superior lamella and ending at peristome. Subcolumellaris emerged and visible in oblique view. Principalis running along ventral-lateral side and anterior end visible through oblique apertural view. Palatal plicae ventral-lateral, five: equally spaced, fourth plica curved most, two middle plicae equally shorter than first and fourth; fifth plica can only be seen when shell is opened. Clausilium plate lateral side, broad, rounded and with hook.

***Genitalia*** (*n* = 2). Atrium (at) small and short; penis (p) almost cylindrical and gradually narrower at transition to epiphallus. Epiphallus (ep) cylindrical and ca. same length and diameter as penis. Penial retractor muscle (pr) attaches proximally to epiphallus. Vas deferens (vd) very thin and shortly bounded at penis (Fig. 14E). Internal sculpture of penis with 3 to 4 large corrugated longitudinal folds (Fig. 14G). Internal sculpture of epiphallus with finely reticulated pattern arranged on two or three large longitudinal folds (Fig. 14H).

Vagina (v) very thin and ~ 1/3 of free oviduct (fo) length. Distal part of pedunculus (dpb) long and enlarged, cylindrical; diverticulum (db) long, enlarged at basal and gradually reduced and becoming slender at terminal, and nearly same length as dpb; proximal part of pedunculus (ppb) short, ~ 3/4 diverticulum length, and bursa copulatrix (bb) small, ovoid (Fig. 14E).

***Radula*.** Central and lateral teeth contain ≥ 23 teeth with half-row formula: central–(lateral)–marginal teeth (1–(10–11)+). Central tooth tricuspid: mesocone large and triangular; ectocone small and located near the base. Lateral teeth bicuspid: endocone large and bluntly pointed tip; ectocone small and pointed tip. Marginal teeth asymmetrically tricuspid starting at approximately tooth number 10 or 11 (Fig. 15E). Most marginal teeth part lost during the cleaning process.

##### Distribution.

This species is presently only known from Shan State, Myanmar.

##### Remarks.

Among all the Myanmar’s *Oospira* species studied herein, this is the only species with a clausilium hook (Fig. 12F), and the clausilium plate is broader than in all other congeners. The presence of the hook in other phaedusinid taxa has been documented and is assumed to provide an advantage in trapping air bubbles to avoid drowning (see Páll-Gergely and Szekeres 2017).

*Oospirasardicola* can be distinguished from *O.shanensis* by having paler glossy yellowish color, wider and less oblique aperture, and less ventricose shell (Szekeres et al. 2021b). This species can clearly be recognized from *O.gouldiana* and *O.magna* by its more ventricose shell, inferior lamella straight ascending and reduced to the peristome and clausilium more rounded, broader, and in lateral position. Moreover, *O.shanensis* has a relatively short male organ, epiphallus larger than the penis, diverticulum narrower to the proximal end, and the internal sculpture is distinctly different from *O.gouldiana* and *O.magna*.

*Oospiramongmitensis*, *O.sardicola*, and *O.shanensis* were recorded from Shan State. All of them are similar in possessing distinct palatal plicae which can be seen through the shell, but different in their pale to dark color, more or less bulging shell and aperture oblique to nearly vertical (Szekeres et al. 2021a, b). Furthermore, *O.sardicola* and *O.mongmitenis* have distinct sutural papillae. These three species; *O.mongmitensis*, *O.sardicola*, and *O.shanensis* from Shan State are notably different from their congeners from southern Myanmar showing anterior end of inferior lamella reduced at peristome.

Our specimens show variation in the development of palatal plicae, especially as the fourth or fifth plicae are horizontal to oblique.

#### ﻿12 Oospira luneainopsis

Taxon classificationAnimaliaStylommatophoraClausiliidae

Man & Panha sp. nov.

B0C5CC33-0563-52C8-ABE8-77501B1AD032

https://zoobank.org/B8866312-31E1-4E9A-993D-6A3E025C8F17

**Figure 16.**
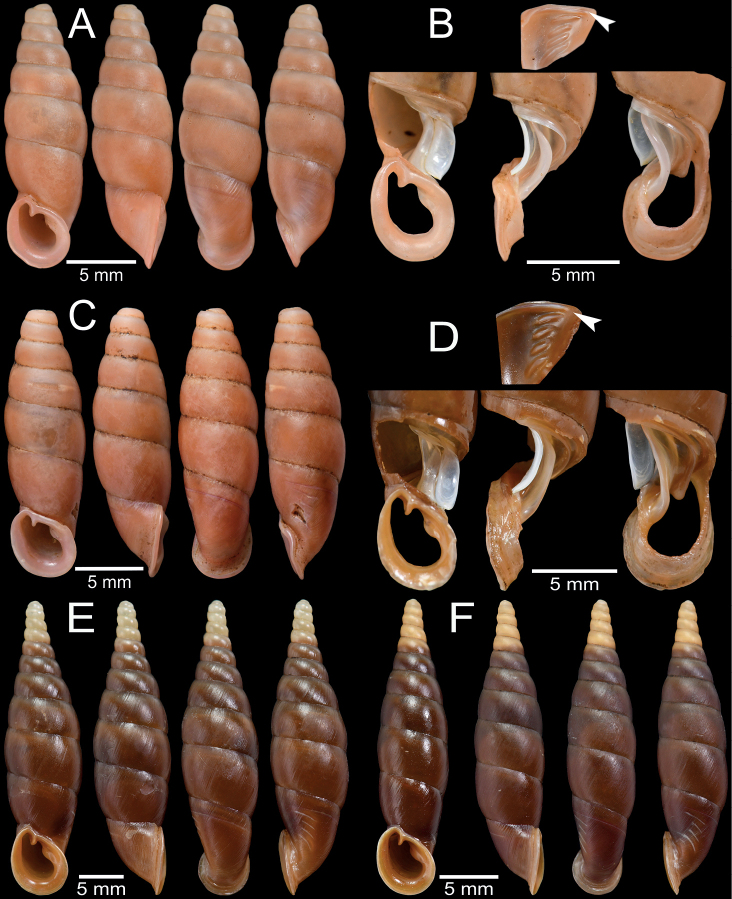
Shell and clausilial apparatus of **A–C***Oospiraluneainopsis* sp. nov. **A** shell of holotype CUMZ 13062 from Zwekabin Mountain, Kayin State **B, C** paratypes CUMZ 13063 from the type locality **B** clausilial apparatus **C** shell and **D–F***Oospirazediopsis* sp. nov. **D** clausilial apparatus of paratype CUMZ 13065 from the type locality **E** shell of holotype CUMZ 13064 from Weibyan Cave, Kayin State and **F** shell of paratype CUMZ 13065 from the type locality.

Figs 16A–C , 18P ; Table 2

##### Type material.

***Holotype***CUMZ 13062 (Fig. 16A). Measurements: shell height 20 mm, shell width 5.8 mm, and 7 whorls. ***Paratypes***CUMZ 13063 (8 shells; Fig. 16B, C), NHMUK 20220504 (2 shells).

##### Type locality.

Limestone outcrop at Zwegabin Mountain, Hpa-an Township, Hpa-an District, Kayin State, Myanmar (16°48'44.5"N, 97°40'25.5"E).

##### Etymology.

The specific name *luneainopsis* is a compound of ‘lun-eain’, which in the Myanmar language refers to a bobbin used for traditional Myanmar weaving, and the Greek suffix -*opsis* meaning ‘having the appearance of or like’. It refers to the bobbin shape of the shell of this species.

**Figure 17.**
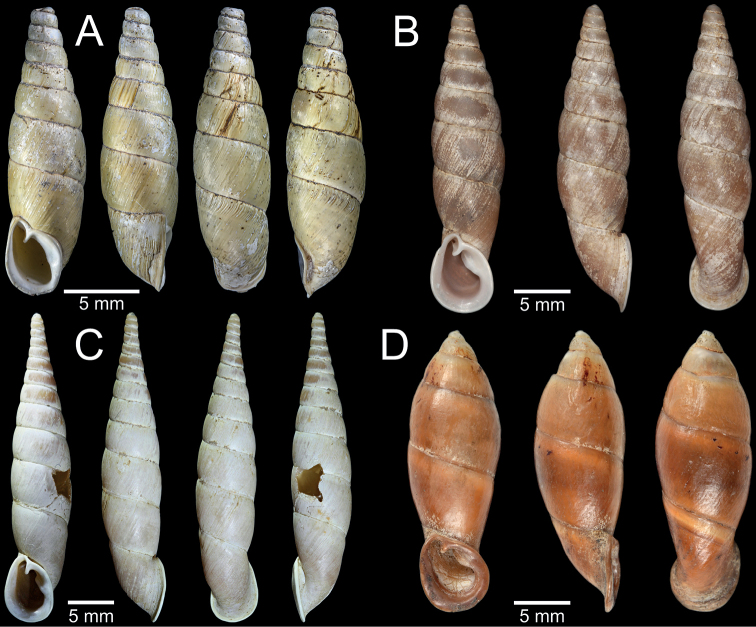
Shell of **A***Oospiradecollata*, paratype ZISP from West Burma, near Pakoku on Mount Victoria. 2200 m a.s.l. **B***Oospirainsignis*, lectotype USNM 117120 from Tavoy, Burmah **C***Oospiramalaisei*, holotype SMNH 3014 from NO-Birma: Pungkataung ca. 3000 ft. and **D***Oospiravespa*, lectotype MCZ 169455 from Tavoy, British Burma. Photo: A Persson, SMNH (**C**).

##### Diagnosis.

Shell ovate-fusiform, decollated with smooth surface and dense striations near suture. Last whorl neck subquadrate; aperture nearly roundish; peristome detached. Inferior lamella straight ascending and reaching at peristome; palatal plicae four, distinct, equally, and parallelly arranged, and superior lamella developed.

##### Description.

Shell ovate-fusiform, decollated, opaque, pinkish brown; spire blunt. Shell surface smooth to with fine growth lines on last whorl, denser near suture and last whorl neck subquadrate; suture impressed and distinct. Whorls 7–8, convex, regularly growing, and bluntly attenuated to apex. Aperture nearly round; peristome detached, thickened, expanded and little reflexed. Superior lamella developed, continuous with spiralis, and slightly low at transition to spiralis. Inferior lamella steeply ascending, distant from superior lamella and ending at peristome. Subcolumellaris emerged and invisible in oblique view. Principalis running along lateral-dorsal side and anterior end visible through oblique apertural view. Palatal plicae lateral, four: first and fourth plicae strong and longest; remaining plicae parallel and equal length; sometimes tiny lowest plica present. Clausilium plate lateral side, narrow and slightly pointed at tip.

##### Distribution.

*Oospiraluneainopsis* sp. nov. is only known from the type locality.

##### Remarks.

No ethanol preserved specimens were available for anatomical study. This species can be distinguished from *O.gouldiana*, *O.magna*, and *O.zediopsis* sp. nov. by its small size, decollated, fewer whorls, roundish aperture, more spiral inferior lamella, and equal palatal plicae. This new species is clearly distinct from *O.decollata* by its spiral inferior lamella visible from aperture, smoother shell, pink color, and more bulging whorls.

Compared to the Vietnamese dextral species, *O.duci* Maassen & Gittenberger, 2007 exhibits thinner and oblique aperture, sinus higher, obviously emerged subcolumellaris, very fine striations and more ventrally unequal palatal plicae than this species.

#### ﻿13 Oospira zediopsis

Taxon classificationAnimaliaStylommatophoraClausiliidae

Man & Panha sp. nov.

69FFBCFA-47E0-5082-AC2E-193DE3A25EDA

https://zoobank.org/FB4630CA-1F88-437F-88C8-8187A9ECAA1E

Figs 16D–F , 19Q ; Table 2

##### Type material.

***Holotype***CUMZ 13064 (Fig. 16E). Measurement: shell height 25 mm, shell width 6.5 mm and 10 whorls. ***Paratypes***CUMZ 13065 (50 shells; Fig. 16D, F), NHMUK 20220508 (2 shells) and JG-C 2884 (2 shells).

##### Type locality.

Weibyan Cave, Hpa-an Township, Hpa-an District, Kayin State, Myanmar (17°13'38.2"N, 97°37'24.0"E).

##### Etymology.

The specific name *zediopsis* is a compound word; ‘zedi’ is one of several terms in the Myanmar language that refer to a stupa or pagoda, and the Greek suffix -*opsis* means ‘having the appearance of or like’. It refers to the Myanmar pagoda-like shell shape of this species.

##### Diagnosis.

Shell fusiform, with four to five white apical whorls, and nearly smooth shell surface. Inferior lamella straight ascending or almost flattened, palatal plicae lateral, equally oblique, superior lamella distinct at transition to spiralis, and clausilium plate lateral side and less bent inwards.

##### Description.

Shell fusiform, opaque, and chestnut-brown color; 4–5 apical whorls white and rapidly attenuated. Shell surface smooth to with very thin growth lines; suture impressed and distinct. Whorls 10–11, convex, regularly growing, and attenuated to apex. Aperture obliquely pear-shaped, and basis rounded; peristome slightly protruded, large, relatively thickened and slightly expanded. Superior lamella developed, connected to spiralis and slightly low at transition to spiralis. Inferior lamella steeply ascending, distant from superior lamella and ending at peristome. Subcolumellaris emerged and invisible in oblique view. Principalis running along lateral-dorsal side and anterior end visible through oblique apertural view. Palatal plicae lateral, four or five: first and fourth plicae strongest and longest; remaining plicae parallel and equal length; fifth plica smallest. Clausilium plate lateral side and narrow.

##### Distribution.

This species is only known from the type locality with plentiful shells collected.

##### Remarks.

No ethanol preserved specimens were available for anatomical study. *Oospiragouldiana* can be distinguished from *O.zediopsis* sp. nov. by its less ventricose shell, denser and stronger striations, more spiral inferior lamella, palatal plicae and clausilium more ventrally positioned, and principalis not visible from the aperture view. Likewise, *O.shanensis* can be differentiated from this new species by wider spacing between spiralis and inferior lamella, anterior end of inferior lamella reduced at peristome, longer palatal plicae, attached peristome, and clausilium wider and with a hook. Furthermore, *O.zediopsis* sp. nov. can be differentiated from *O.insignis* by its more attenuated spire, narrower and more oblique aperture, shorter and more equal palatal plicae.

## ﻿Discussion

**Figure 18.**
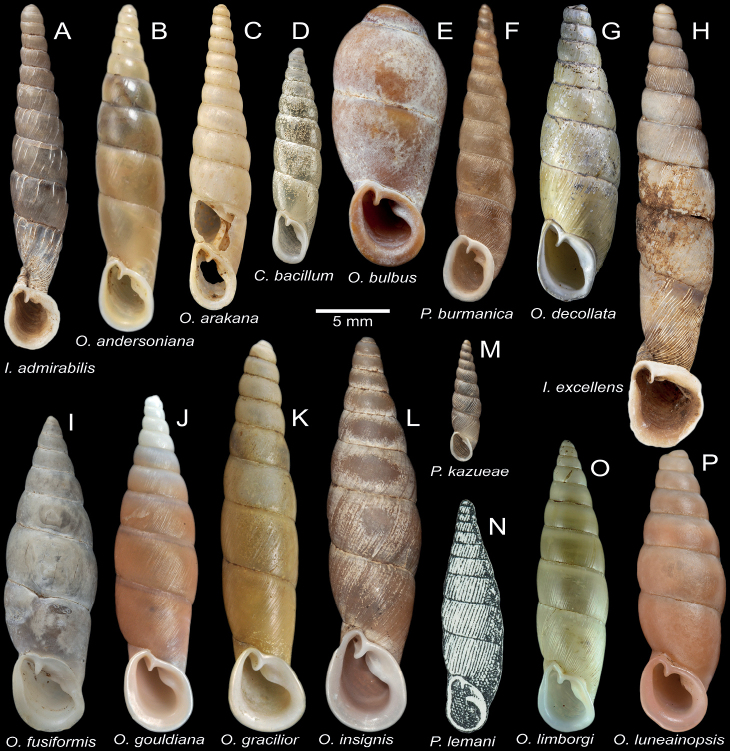
Synoptic view of the clausiliid species recorded from Myanmar **A***Indoneniaadmirabilis*, holotype NHMUK 20200185 **B***Oospiraandersoniana*, specimen CUMZ 13054 **C***Oospiraarakana*, lectotype NHMUK 1888.12.4.1037 **D***Cylindrophaedusabacillum*, specimen NHMUK 1906.2.2.345 **E***Oospirabulbus*, possible syntype NHMUK 1903.7.1.1278 **F***Phaedusaburmanica*, holotype NHMUK 1888.12.4.1058 **G***Oospiradecollata*, paratype ZISP**H***Indoneniaexcellens*, specimen NHMUK 20200189 **I***Oospirafusiformis*, holotype NHMUK 1906.2.2.380 **J***Oospiragouldiana*, lectotype NHMUK 196548 **K***Oospiragracilior*, lectotype NHMUK 1907.12.30.243 **L***Oospirainsignis*, lectotype USNM 117120 **M***Phaedusakazueae*, holotype NHMUK 20200187 **N***Phaedusalemani* (after Gude, 1914: fig. 107) **O***Oospiralimborgi*, holotype NHMUK 1903.7.1.1266, and **P***Oospiraluneainopsis* sp. nov., holotype CUMZ 13062.

**Figure 19.**
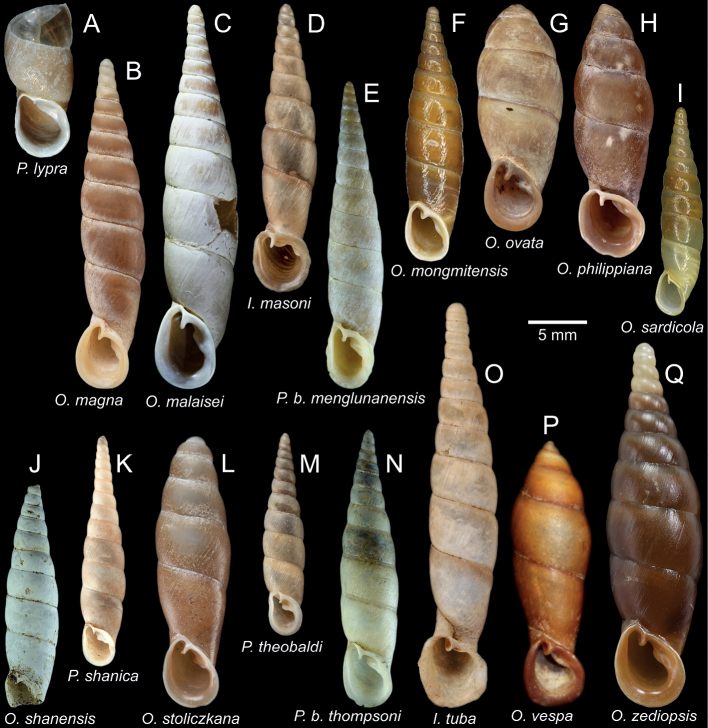
Synoptic view of the clausiliid species recorded from Myanmar **A***Phaedusalypra*, syntype MNHN-IM-2000-2502-7 **B***Oospiramagna*, lectotype NHMUK 1901.3.17.156 **C***Oospiramalaisei*, holotype SMNH 3014 **D***Indoneniamasoni*, lectotype NHMUK 1888.12.4.1034 **E***Phaedusabockimenglunanensis*, specimen NHMUK 1903.7.1.3652 **F***Oospiramongmitensis*, holotype UF 117571 (after Szekeres et al. 2021b: fig. 1d) **G***Oospiraovata*, lectotype NHMUK 1906.2.2.378 **H***Oospiraphilippiana*, possible syntype SMF 62294/1 **I***Oospirasardicola*, holotype UF 117570 (after Szekeres et al. 2021b: fig. 2c) **J***Oospirashanensis*, holotype NHMUK 1903.7.1.3544 **K***Phaedusashanica*, lectotype SMF 62260 **L***Oospirastoliczkana*, holotype NHMUK 1888.12.4.1031 **M***Phaedusatheobaldi*, lectotype NHMUK 1888.12.4.1056 **N***Phaedusabockithompsoni*, specimen NHMUK 1903.7.1.1163 **O***Indoneniatuba*, lectotype NHMUK 1907.12.30.241 **P***Oospiravespa*, paralectotype MCZ 169456 and **Q***Oospirazediopsis* sp. nov., holotype CUMZ 13064. Photo: P. Maestrati, MNHN (**A**), A Persson, SMNH (**C**).

Among the 33 clausiliid taxa recorded from Myanmar, 13 species are redescribed here based on the shell, genitalia, and radular morphologies, including two new species. The remaining 20 species are known from literature, and images of the type specimens, authenticated specimens or original illustrations are provided for further comparison (Figs 18, 19). All examined clausiliid genitalia in Myanmar show simple male organs (penis, epiphallus, penial retractor muscle, and vas deferens) and these differ mainly in the length of penis, diverticulum, and pedunculus. Internally, the epiphallus is more variable with two or three patterns on the surface, and different numbers of simple longitudinal folds in penis. The genitalia of the recently reported *Phaedusabhutanensis* Nordsieck, 1974 from Bhutan is similar to the Myanmar *Phaedusa* species examined in this study in having a distinctly long diverticulum, pedunculus and developed male organs (see Gittenberger et al. 2019: figs 27b, 28, 29). Overall, the external shell morphology, including shape, size, whorl number, and color is useful in recognizing the Myanmar clausiliids at first glance.

In Myanmar, *Oospira* is one the most diverse stylommatophoran genera. It comprises 20 species of which ten are redescribed herein, including the type species, *O.philippiana*. Based on the shell, clausilial apparatus and genital morphology, the genus is generally divided into two groups: (1) short and ovate fusiform shell group (including type species), and (2) long and slender fusiform shell group. The first group tends to have palatal plicae reduced towards the anterior, inferior lamella straight ascending and superior lamella distinct from spiralis at its transition point, a well-developed penis, diverticulum, and proximal of pedunculus almost similar in length, internal wall of epiphallus with equally and obliquely arranged papillae, and internal wall of penis consists of two shapes: oblique V-shaped near atrium and transitioning to longitudinal folds near epiphallus. The second group possess palatal plicae that are not reduced towards the anterior, inferior lamella more spirally ascending (S-shape) and superior lamella indistinct from spiralis at it transition point, slender to narrowly cylindrical penis, generally diverticulum longer than proximal of pedunculus in length, internal wall of epiphallus with irregularly arranged papillae, and internal wall of penis consists of longitudinal folds throughout the entire chamber. Additionally, the central tooth of group (1) has a more rounded cusp than group (2), which has a triangular shape and pointed cusp. These differences in shell and clausilial apparatus between the two groups rather agree with the diagnostic characters of the *Pseudonenia* Boettger, 1877 (see Gude 1914). However, further supporting evidence and the morphological and anatomical information of its type species are needed to elucidate and resolve the taxonomy of these groups.

Although the clausiliids seem evenly distributed throughout Myanmar, *Phaedusa* is distributed in the northeast to southwest, while *Oospira* occurs widely along the northernmost to the southernmost parts of the country (Fig. 1; Table 1). Based on our surveys, the southern part of Myanmar, including Kayin and Mon States and the Tanintharyi Region, has the largest number of species recorded, and seven species are assumed to be endemic to this area. Allopatric speciation caused by massive and isolated limestone karsts in the topography of the Salween River Basin and the Tenasserim Range possibly plays an important role in the high endemicity of clausiliids. Recent studies on streptaxid and helicarionid snails have shown a similar pattern (Sutcharit et al. 2020a; Man et al. 2022; Pholyotha et al. 2022). However, alternative forces of sympatric speciation and resource partitioning cannot be ignored. For example, *O.andersoniana* and *O.stoliczkana* in Tanintharyi are sympatric based on this study. The lower abundance of one species compared to the co-occurring species may reflect the high level of competition (Table 1).

Despite the numbers of the Myanmar clausiliids are increasing, the taxonomic placements of some species are still questionable, for example, *Oospiragouldiana* and *O.insignis* which are morphologically highly variable and have overlapping distributions. Previous studies have attempted to reveal their differentiation, but this remains ambiguous (Stoliczka 1872; Gude 1914; Szekeres et al. 2021a, b). This work, therefore, provides baseline information for future studies, especially with respect to the phylogenetic interpretation of the short and ovate shell group that is endemic in Mon State and the long and slender shell group, with its wider distribution from Shan State to Kayin State and the Tanintharyi Region.

## ﻿Alphabetical list of additional clausiliid taxa recorded from Myanmar

Below is an alphabetical list of 20 clausiliid species and subspecies belonging to the genera *Cylindrophaedusa*, *Indonenia* Ehrmann, 1927, *Oospira*, and *Phaedusa* that have been reported to occur in Myanmar, but that were not found during this study. The usages of each taxon name are provided in Grego et al. (2021) and Szekeres et al. (2021a, b). The original combination of the taxon name with reference to pages, plates, and/or figures that made the names available is mentioned. The type locality mentioned in the original publication and the distribution record in Myanmar is included. If possible, the modern name and/or regional names of the type locality are provided in square brackets. In addition, the unique name-bearing types (syntype, holotype, and lectotype) are illustrated; only for those that were unavailable or could not be located, the paratypes or paralectotypes or a representative specimen are given. Where necessary, remarks are given on the status of its nominal taxon, type specimens, and other necessary information.

**1 *Cylindrophaedusabacillum* (Hanley & Theobald, 1870)**

Fig. 18D

*Clausiliabacillum* Hanley & Theobald, 1870: 12, pl. 24, fig. 1. Type locality: “Nanclai, Khasi Hills”. Sowerby 1875: *Clausilia* pl. 6, species 48.

*Clausilia* (? *Medora*) *bacillum*–Blanford 1872: 200, pl. 9, fig. 3.

Clausilia (Phaedusa) bacillum–Tapparone-Canefri 1889: 328, 329. Gude 1914: 305.

*Hemiphaedusabacillum*–Nordsieck 1973: 72, pl. 3, fig. 10.

Cylindrophaedusa (Montiphaedusa) bacillum–Szekeres et al. 2021a: 161, 162, fig. 3e.

**Distribution.** In Myanmar, this species is known from Schegoo (presumably Shwegu), Bhamo City in Kachin State; no further materials have been reported from Myanmar after its mention in Tapparone-Canefri (1889) and Gude (1914).

**2 *Oospiraarakana* (Stoliczka, 1872)**

Fig. 18C

*Clausiliaarakana* Stoliczka, 1872: 210, pl. 9, fig. 20. Type locality: “Mai-i in provincia Sandoway” [Mwa-ywa, Thandwe Township, Rakhine State]. Hanley and Theobald 1874: 48, pl. 118. figs 8, 9. Sowerby 1875: *Clausilia* pl. 9, species 78.

Clausilia (Phaedusa) arakana–Gude 1914: 312.

*Oospiraarakana*–Nordsieck 1974: 47, fig. 8, pl. 2, fig. 8. Szekeres et al. 2021a: 165, fig. 4d.

**Distribution.** This species is probably endemic to Rakhine State, Myanmar.

**3 *Oospiradecollata* (Likharev, 1962)**

Figs 17A, 18G

*Pseudoneniadecollata* Likharev, 1962: 11–13, figs 1, 2. Type locality: “Nat Ma Taung, Chin State, Myanmar”.

Oospira (Oospira) decollata–Nordsieck 2002b: 86. Nordsieck 2007: 23.

*Oospiradecollatadecollata*–Szekeres et al. 2021a: 168.

**Distribution.** This species was recorded from Nat Ma Taung, also called Victoria Mountain, Chin State, and as such it is the westernmost clausiliid species recorded in Myanmar. Another subspecies, *O.decollatamuspratti* Grego & Szekeres, 2021 was recently reported from Naga Hills, India (Szekeres et al. 2021a).

**4 *Oospirafusiformis* (Blanford, 1865)**

Fig. 18I

*Clausiliafusiformis* Blanford, 1865: 80. Type locality: “Arakan Hill, west of Henzada” [Rakhine State or west of Hinthada Township, Ayeyarwady Region]. Hanley and Theobald 1870: 12, pl. 24, fig. 6. Sowerby 1875: *Clausilia* pl. 7, species 62.

Clausilia (Phaedusa) fusiformis–Gude 1914: 321.

*Oospirafusiformis*–Szekeres et al. 2021a: 169–171, fig. 6b, c.

**Distribution.** In Myanmar, this species is known from Rakhine State and Ayeyarwady Region, and also reported from the Bago Region (Gude 1914). In addition, *O.fusiformis* was recorded from ‘Zwekabin Hill near Mawlamyine’, which the current administration defines as Zwekabin Mountain in Hpa-an District, Kayin State; however, it was noted as dubious by Szekeres et al. (2021a: 171).

**Remarks.**Blanford (1865) described this species based on a single specimen and also noted that the examined specimen was an immature shell because the shell was thin, and the peristome not continuous. Later, Blanford (1872) provided a re-description based on a fully adult specimen received from W. Theobald that had a thickened, expanded, and continuous peristome.

The specimen identified as *Oospirainsignis*Szekeres et al. (2021a: 172, fig. 7a) is similar to *O.fusiformis* (Fig. 18I) in terms of shell morphology. Blanford (1872) also knew these similarities and suggested uniting the two species; however, further evidence from the shell and genitalia from topotypic specimens will be necessary to verify their species status.

**5 *Oospiragracilior* (Hanley & Theobald, 1870)**

Fig. 18K

Clausiliainsignisvar.gracilior Hanley & Theobald, 1870: 12, pl. 24, fig. 3. Type locality: “Burma, Moulmain” [Mawlamyine Township, Mon State, Myanmar].

Clausilia (Phaedusa) gracilior–Gude 1914: 318, 319, fig. 110.

**Distribution.** In Myanmar, this species is known from Mon State.

**Remarks.** Hanley and Theobald (1870) introduced this species as a variety of *O.insignis* but did not provide a description. Later Gude (1914) treated it as a separate species and made a complete re-description. We treat this taxon as a distinct species, following Gude (1914), because it clearly differs from *O.insignis* s.s. in being less ventricose, with blunt spire, and the aperture nearly triangular that is broader at its basis; however, no materials for this species have been recollected since the original description.

**6 *Oospirainsignis* (Gould, 1843)**

Figs 17B, 18L

*Clausiliainsignis* Gould, 1843: 140. Type locality: “Tavoy, British Burma” [Dawei, Tanintharyi Region, Myanmar]. Gould 1844: 458, pl. 24, fig. 8. Johnson 1964: 92, pl. 36, fig. 9.

Clausilia (Phaedusa) insignis–Gude 1914: 319.

*Oospirainsignis*–Nordsieck 2021: 6, pl. 1, fig. 6. Szekeres et al. 2021a: 172, fig. 7a. Szekeres et al. 2021b: 41, fig. 1b.

**Distribution.** In Myanmar, this species is originally known from Dawei, Tanintharyi Region. Subsequent records were from Mon and Kayin states in Myanmar, and the range was further extended to include Tak and Kanchanaburi provinces in Thailand (Gude 1914; Szekeres et al. 2021a, b).

**Remarks.***Oospirainsignis* was the first clausiliid discovered from Myanmar, introduced by A.A. Gould based on specimens from the American missionary and naturalist Francis Mason (1799–1874). In the original description, Gould (1843) did not clearly state the number of examined specimens or the type specimen deposition. Later, Gould (1844) re-described this species with an illustration. When Johnson (1964) catalogued Gould’s type specimen, he clearly designated the lectotype USNM 117120 as the unique name-bearing type. However, Nordsieck (2007: pl. 1, fig. 1) and Nordsieck (2021: pl. 1, fig. 6) illustrated a specimen BM(NH) 1996183 [=NHMUK] from the Cuming collection and used the term ‘lectotype’, but this seems invalid; recently, Szekeres et al. (2021a) designated this specimen as the lectotype. This specimen lot NHMUK 1996183a ex. Cuming collection consisted of three shells with no clear evidence that they originated from Gould’s collection. Although Johnson (1964) catalogued Gould’s type specimens in the Museum of Comparative Zoology, Harvard University, he stated that some unlocated types might presumably be deposited in the NHM, London, because Gould brought some specimens to compare to the Cuming collection. Presumably, Nordsieck (2007) and Szekeres et al. (2021a) may have overlooked the previous lectotype designated by Johnson (1964), which clearly stated that the specimen USNM 117120 ex. Lea collection ex. Gould’s collection consists of a single shell as the lectotype. Therefore, the USNM 117120 specimen is the sole name-bearing type of this species. The latter restriction of the lectotype by Nordsieck (2007) and Szekeres et al. (2021a) is invalid (ICZN, 1999: Art. 74.1). Additionally, the specimen NHMUK 1996183 is potentially a part of Gould’s type series and is here recognized as the possible paralectotype.

**7 *Oospiralimborgi* Grego & Szekeres, 2021**

Fig. 18O

*Oospiralimborgi* Grego & Szekeres in Szekeres et al. 2021a: 172–174, fig. 7c. Type locality: “Tanintharyi Region, Myanmar”.

**Distribution.** In Myanmar, this species is known only from Tanintharyi Region.

**8 *Oospiramalaisei* Nordsieck, 1973**

Figs 17C, 19C

*Oospiramalaisei* Nordsieck, 1973: 81, 82, pl. 3, fig. 3. Type locality: “Punkataung bei Myitkyina (3000 ft), NO-Birma” [near Punkataung, Myintkyina Township, Kachin State, Myanmar]. Szekeres et al. 2021b: 41–43.

**Distribution.** This species was introduced from Kachin State, and no subsequent materials have been recorded from Myanmar. Recently, provisionally classified specimens belonging to this species were reported from Mae Hong Son Province in Thailand (Szekeres et al. 2021b). In addition, it was further reported from Yunnan, China, and included in a molecular analysis (Hwang et al. 2022).

**9 *Oospiramongmitensis* Grego & Szekeres, 2021**

Fig. 19F

*Oospiramongmitensis* Grego & Szekeres in Szekeres et al. 2021b: 43, fig. 1d. Type locality: “Momeit” [Mongmit, Shan State, Myanmar].

**Distribution.** In Myanmar, this species is only known from Shan State.

**10 *Oospirasardicola* Grego & Szekeres, 2021**

Fig. 19I

*Oospirasardicola* Grego & Szekeres in Szekeres et al. 2021b: 46, 47, fig. 2c. Type locality: “Ruby Mines, Burmah” [around Mongmit, Shan State, Myanmar].

**Distribution.** In Myanmar, this species is only known from Shan State.

**11 *Oospiravespa* (Gould, 1856)**

Figs 17D, 19P

*Clausiliavespa* Gould, 1856: 13. Type locality: “Tavoy” [Dawei, Tanintharyi Region, Myanmar].

Clausilia (Oospira) vespa–Gude 1914: 336, fig. 116.

*Oospiravespa*–Szekeres et al. 2021a: 180, fig. 9c.

**Distribution.** In Myanmar, this species is known from Tanintharyi Region and Salween Valley (Sykes, 1893).

**Remarks.***Oospiravespa* is the second clausiliid species introduced by Gould (1856). It was described based on a collection by the American naturalist, F. Mason, from Tavoy, British Burma. In the original publication, Gould (1856) provided only a brief description, without the arrangement and morphology of plicae, illustration, or number of examined specimens. Later, Johnson (1964) catalogued Gould’s type specimens and found two specimens, which validly designated a specimen lot MCZ 169455 as the lectotype, which is figured herein (Fig. 17D).

While describing *Oospirastoliczkana* from Myanmar, Sykes (1893) re-described *O.vespa* based on a specimen from ‘Salwin Valley’. Then, it was re-described based on Gould’s original type series (Gude 1914). Between the two species, *O.vespa* can be distinguished from *O.stoliczkana* by having a more acute spire, and only five or six palatal plicae. In contrast, *O.stoliczkana* possesses a blunt spire, and 8 to 10 palatal plicae.

**12 *Phaedusakazueae* Hunyadi & Szekeres, 2021**

Fig. 18M

*Phaedusakazueae* Hunyadi & Szekeres in Grego et al. 2021: 25–27, fig. 5b. Type locality: “Montawa Cave, SW of Taunggyi, Shan State, Myanmar”.

**Distribution.** In Myanmar, this species is only known from Shan State.

**13 *Phaedusalemani* (Gude, 1914)**

Fig. 18N

Clausilia (Phaedusa) lemani Gude, 1914: 313, 314, fig. 107. Type locality: “Arakan, Burma” [Rakhine State, Myanmar].

**Distribution.** In Myanmar, this species is only known from Rakhine State and no further materials have been mentioned to date.

**14 *Phaedusalypra* (Mabille, 1887)**

Fig. 19A

*Clausilialypra* Mabille, 1887: 117. Type locality: “Tonkin” [Vietnam].

Phaedusa (Phaedusa) lypra–Nordsieck 2002b: 88.

*Phaedusalypra*–Grego et al. 2021: 27, fig. 5c. Szekeres et al. 2021a: 183, fig. 10e, f.

**Distribution.** In Myanmar, this species is known from Shan State and Mandalay Region (Grego et al. 2021). *Phaedusalypra* has a wide geographical distribution, ranging from China to Thailand, Vietnam, and India (Szekeres et al. 2021a).

**15 *Phaedusatheobaldi* (Blanford, 1872)**

Fig. 19M

*Clausilia* (? *Medora*) *theobaldi* Blanford, 1872: 201, pl. 9, fig. 5. Type locality: “Tonghu in provincia Barmana” [Taungoo Township, Bago Region, Myanmar].

Clausilia (Phaedusa) theobaldi–Gude 1914: 310.

*Phaedusatheobaldi*–Nordsieck 1974: 45, fig. 4, pl. 2, figs 1, 2. Grego et al. 2021: 27, fig. 5d. Szekeres et al. 2021a: 185, fig. 11c–e.

**Distribution.** In Myanmar, this species was first known from Bago Region. Later, the range was expanded records from Rakhine and Kayah States in Myanmar and from Thailand (Gude 1914; Grego et al. 2021; Szekeres et al. 2021b).

**16 *Phaedusabockithompsoni* Grego & Szekeres, 2021**

Fig. 19N

*Phaedusabockithompsoni* Grego & Szekeres in Szekeres et al. 2021b: 46, fig. 2d. Type locality: “Doi Tung, Chiang Rai Province, Thailand”.

**Distribution.** In Myanmar, a single specimen was recorded in Shan State (Szekeres et al. 2021a). This subspecies was originally described from northern Thailand with several specimens.

**17 *Indoneniaadmirabilis* Grego & Szekeres, 2021**

Fig. 18A

*Indoneniaadmirabilis* Grego & Szekeres in Grego et al. 2021: 22, figs 2b, 3, 4. Type locality: “Phruno River Cave, Maw Ti Do, Hpruso District, Kayah State, Myanmar”. Szekeres et al. 2021a: 157, fig. 2a.

**Distribution.** In Myanmar, this species is only known from Kayah State.

**18 *Indoneniaexcellens* (Nordsieck, 2002)**

Fig. 18H

Tropidauchenia (Indonenia) excellens Nordsieck, 2002a: 16, fig. 6. Type locality: “Karen Mountains, 1200–1300m” [Kayin or Kayah State, Myanmar].

*Indoneniaexcellens*–Nordsieck 2007: 37, pl. 6, fig. 4. Szekeres et al. 2021a: 157, fig. 2b, c.

**Distribution.** In Myanmar, this species is known from Kayin or Kayah States, originally given type locality was uncertain. Recently, it was reported from Shan State (Grego et al. 2021).

**19 *Indoneniamasoni* (Theobald, 1864)**

Fig. 19D

*Clausiliamasoni* Theobald, 1864: 246. Type locality: “Tonghu” [Taungoo Township, Bago Region, Myanmar]. Hanley and Theobald 1870: 12, pl. 24, fig. 8. Sowerby 1875: *Clausilia* pl. 8, species 69.

*Clausilia* (? *Nenia*) *masoni*–Blanford 1872: 206, pl. 9, fig. 18.

Clausilia (Garnieria) masoni–Gude 1914: 331.

*Indoneniamasoni*–Nordsieck 2007: 37. Szekeres et al. 2021a: 157, fig. 2d.

**Distribution.** In Myanmar, this species is only known from Bago Region.

**20 *Indoneniatuba* (Hanley, 1868)**

Fig. 19O

*Clausiliatuba* Hanley, 1868: 343. Type locality: “Shan, province” [Shan State, Myanmar]. Hanley and Theobald 1870: 12, pl. 24, fig. 9. Sowerby 1875: *Clausilia* pl. 8, species 72.

Clausilia (Garnieria) tuba–Gude 1914: 331, fig. 115.

*Indoneniatuba*–Szekeres et al. 2021a: 157–159, fig. 2e, f.

**Distribution.** In Myanmar, this species is known from Shan State and the Upper Salween Valley (Hanley 1868).

## Supplementary Material

XML Treatment for Phaedusa

XML Treatment for Phaedusa shanica

XML Treatment for Phaedusa burmanica

XML Treatment for Phaedusa bocki menglunanensis

XML Treatment for Oospira

XML Treatment for Oospira philippiana

XML Treatment for Oospira bulbus

XML Treatment for Oospira ovata

XML Treatment for Oospira stoliczkana

XML Treatment for Oospira gouldiana

XML Treatment for Oospira andersoniana

XML Treatment for Oospira magna

XML Treatment for Oospira shanensis

XML Treatment for Oospira luneainopsis

XML Treatment for Oospira zediopsis
